# Understanding the Impact of Different Modes of Information Provision on Preferences for a Newborn Bloodspot Screening Program in the United Kingdom

**DOI:** 10.1177/23814683241232935

**Published:** 2024-03-04

**Authors:** Stuart J. Wright, Caroline M. Vass, Fiona Ulph, Katherine Payne

**Affiliations:** Manchester Centre for Health Economics, Division of Population Health, Health Service Research and Primary Care, The University of Manchester, Manchester, UK; Manchester Centre for Health Economics, Division of Population Health, Health Service Research and Primary Care, The University of Manchester, Manchester, UK; RTI Health Solutions, The Pavilion, Towers Business Park, Didsbury, Manchester, UK; Division of Psychology and Mental Health, The University of Manchester, Manchester, UK; Manchester Centre for Health Economics, Division of Population Health, Health Service Research and Primary Care, The University of Manchester, Manchester, UK

**Keywords:** newborn bloodspot (or blood spot) screening, information provision, discrete choice experiment, patient choice

## Abstract

**Introduction.** This study aimed to understand the impact of alternative modes of information provision on the stated preferences of a sample of the public for attributes of newborn bloodspot screening (NBS) in the United Kingdom. **Methods.** An online discrete choice experiment survey was designed using 4 attributes to describe NBS (effect of treatment on the condition, time to receive results, whether the bloodspot is stored, false-positive rate). Survey respondents were randomized to 1 of 2 survey versions presenting the background training materials using text from a leaflet (leaflet version) or an animation (animation version). Heteroskedastic conditional logistic regression was used to estimate the effect of mode of information provision on error variance. **Results.** The survey was completed by 1,000 respondents (leaflet = 525; animation = 475). Preferences for the attributes in the DCE were the same in both groups, but the group receiving the animation version had 9% less error variance in their responses. Respondents completing the animation version gave higher ratings compared with the leaflet version in terms of ease of perceived understanding. Subgroup analysis suggested that the animation was particularly effective at reducing error variance for women (20%), people with previous children (16.5%), and people between the ages of 35 and 45 y (11.8%). **Limitations.** This study used simple DCE with 4 attributes, and the results may vary for more complex choice questions. **Conclusion.** This study provides evidence that that supplementing the information package offered to parents choosing to take part in NBS with an animation may aid them their decision making. Further research would be needed to test the animation in the health system. **Implications.** Researchers designing DCE should carefully consider the design of their training materials to improve the quality of data collected.

**Highlights:**

- Prior to completing a discrete choice experiment about newborn bloodspot screening, respondents were shown information using either a leaflet-based or animated format.
- Respondents receiving information using an animation version reported that the information was slightly easier to understand and exhibited 9% less error variance in expressing their preferences for a newborn screening program.
- Using the animation version to present information appeared to have a larger impact in reducing the error variance of responses for specific respondents including women, individuals with children, individuals between the ages of 35 and 45 y, and individuals educated to degree level.

The need for relevant information provided in an accessible format spans the delivery of many types of health care interventions and national programs. One example of a national program in which information provision is a fundamental requirement is the newborn bloodspot screening (NBS) program in the United Kingdom.^1^ In the United Kingdom, the NBS program involves taking a drop of blood from the heel of a newborn baby, usually when they are less than 1 wk old.^2^ The test occurs early in life, with the aim of identifying and treating 9 serious conditions as early as possible to improve the child’s prognosis. These conditions are phenylketonuria, cystic fibrosis, sickle cell disease, congenital hypothyroidism, medium-chain acyl CoA dehydrogenase deficiency, maple syrup urine disease, isovaleric acidaemia, glutaric acidaemia type1, and homocystinuria.

The parental decision about whether to allow their infant to participate in the bloodspot screening program is a key choice at the start of a child’s life.^1^ Effective information provision is seen as core to making a “good” decision and having a choice.^3,4^ There is also evidence that information provision may be valued for minimizing distress, particularly in situations in which further testing is required after the initial screening test and/or diagnoses are given.^5^ Parents potentially participating in an NBS program must be clear about the potential harms of NBS (distress following unexpected diagnosis, concerns about carrier status) as well as the potential benefits.

Publicly available documents for health professionals clearly outline what information should be given to parents about an NBS program. In the United Kingdome, this includes making sure the parents have the information that they are giving consent for the sample to be taken, sample to be analyzed in the laboratory and used for quality assurance, laboratory to send the results to the child health records department, results being stored on the child health information system, potential identification of their baby as a “carrier” of sickle cell disease or cystic fibrosis, and referral to specialist services if a result is positive. Parents must also be aware they are giving consent for the blood spot card being stored for a minimum period by the laboratory and their baby’s anonymized data being used for research studies.^6^ Up until early 2019, alongside verbal provision of information by a midwife, health professionals were also guided to provide an information booklet called *Screening Tests for You and Your Baby*.^7^ From May 2019, the United Kingdom moved to offer the information from this booklet online, alongside an animation embedded in material on a separate page for parents on screening tests in pregnancy.^8,9^

In active NBS programs across the world, the most frequent modes of information provision are verbal from health care professional, leaflets, or a combination of a leaflet supporting verbal communication.^10–12^ With the observed constraints limiting the time available for health professionals to provide information, leaflets tend to be the predominant mode of information provision for parents making the informed choice about NBS.^1^ There are concerns raised about the use of leaflets, such as poor literacy levels and evidence that information coverage in leaflets is often not comprehensive and has been suggested to present a biased picture.^13,14^ Crucially, there is substantial evidence to support the assumption that parents do not read leaflets, and their efficacy in providing useful information is limited in the context of making an informed choice for NBS.^1,4,10,12,15–17^ Some parents may use the Internet as an additional source of information gathering. The possibility of using non–paper-based alternative modes of information provision, such as videos, has generated mixed findings.^18^ Yang et al.^19^ reported that a 10-min video provided before birth resulted in parents being more likely to engage in appropriate postresult behaviors and also to retain information. However, others reported that mothers in their sample did not find videos to be an effective communication model.^17^

Across countries, there are substantial differences in the type and number of conditions included in an NBS program and differences in opinion about the appropriate model of consent. Despite an NBS program being in place for almost 50 y in some countries, an enduring challenge is the requirement for an evidence-based and effective mode of information provision.^1^ Previous stated preference studies have been used to explore participants’ preferences for the attributes of an NBS or the way in which information is provided as part of the NBS.^20–23^ However, no research has been conducted to determine the potential impact of using different modes of information of participants’ preferences and level of perceived understanding about an NBS. Within this context, this study aimed to understand the stated preferences of a sample of the public for attributes of an NBS and whether the mode of information provision affected these preferences or the error exhibited by participants in expressing their preferences. This study also sought to explore whether the types of information resulted in different levels of improvement or decline in error variance in different subgroups of the sample. Differences in participants’ reported ability to make a decision about screening having received NBS information in different formats were also explored.

To address these aims, 4 research questions were posed. The first research question was, What are they key drivers of preferences for participation in NBS in the United Kingdom? The second research question being addressed was, If, and how, does the mode of information provision affect stated preferences for NBS? The third research question was, Is there evidence that different modes of information provision may be particularly beneficial for different subgroups of the population? The final research question was, Do different modes of information provision affect participant’s self-reported understanding of the information and the ease of making a decision about screening?

## Methods

A discrete choice experiment (DCE), embedded in an online survey, was designed in accordance with published criteria and is reported in line with published guidance.^24–27^ Two versions of the online survey were created that differed in the mode of information provision about NBS provided in the training materials at the start of the survey. The same DCE design and background questions were used in each version of the online survey. Ethical approval for this study was granted by University of Manchester’s Proportionate University Research Ethics Committee (2020-7102-12847).

### Conceptualizing the Choice Question

**Figure 1:**
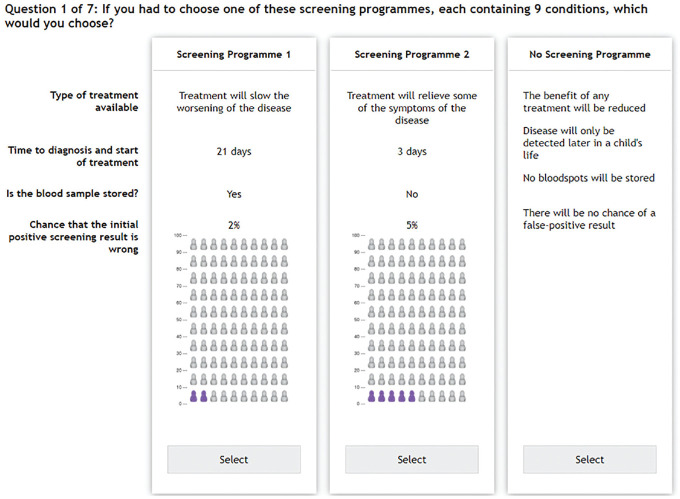
Example choice set.

The choice question defined in the DCE was framed to understand the stated preferences of a sample of individuals for NBS in the UK setting. The choice question posed to each respondent was, If you had to choose 1 of these screening programs, each containing 9 conditions, which would you choose? Each choice set (see Figure 1) contained 3 alternatives: screening program 1, screening program 2, and no screening. No screening represented the alternative to allow opting out of a NBS program.

### Defining Attributes and Levels

The attributes to describe the alternative NBS programs, and the opt-out alternative, used in the DCE (see Table 1), were identified from a published systematic review of studies eliciting preferences for antenatal and NBS programs and qualitative interviews with parents and midwives conducted as part of a larger research study.^1,28^ Four published stated preference surveys were identified that quantified preferences for a NBS program.^20–22,29^ The candidate attributes selected from these 4 published DCEs, and the qualitative interviews, were the type of treatment available for children with inherited diseases that are detected early by screening, the time from the screening test to diagnosis and the start of treatment, whether the bloodspot was stored or not, and the false-positive rate of screening. Levels for these attributes were assigned based on the potential values for each level seen in current implementations of NBS programs.^1^

**Table 1:** Attributes and Levels Used in the Choice Sets

Attribute	Levels	Justification for Inclusion	Coding of Variable in Design and Analysis
Type of treatment available	Treatment will stop the disease from getting worse. Treatment will slow the worsening of the disease Treatment will relieve some of the symptoms of the disease.	Globally, different NBS programs include a different range of conditions. In the United Kingdom, conditions are added only if early treatment can significantly improve outcomes for patients. In some countries, conditions are also included to provide early diagnosis even when treatment options are more limited.	Effects coded
Time to diagnosis and start of treatment	3 d 7 d 14 d 21 d	For many inherited diseases, treatment must be started quickly to prevent damage. In addition, long waits for test results may increase parental anxiety.	Continuous
Is the bloodspot sample stored?	Yes No	Previous research has shown that many parents are not aware that a bloodspot is stored for future research use.	Effects coded
Chance that the initial positive screening result is wrong	1% 2% 5% 10%	False-positive results from NBS may cause parental anxiety, and the number of such results may increase as the number of included conditions increases.	Continuous

NBS, newborn bloodspot screening.

### Creating the Experimental Design

The experimental design of this DCE aimed to minimize the potential effect on the error variance of respondents’ choices by reducing the cognitive burden of the survey. Therefore, a simple design with a relatively small number of choice sets was created.^30,31^ A D-efficient design, using NGene, created the choice sets.^32^ D-efficiency involves minimizing the inverse of the variance-covariance matrix in a maximum likelihood estimation.^33^ Small prior values for the coefficients were included to indicate the expected ordering of the levels as a pragmatic approach to limit the number of dominant choice profiles appearing in the design. Each respondent was randomized to answer 7 choice questions. Of the 24 available choice sets, 4 blocks each containing 6 questions were created. An additional question was created and added to each of the 4 blocks as a test for internal validity as a dominance check. The validity check featured a choice set in which 1 choice profile featured levels that were better or equal to the levels in the alternative profile. The method proposed by Tervonen et al.^34^ was used to predict the failure rate of the dominance choice question using the results of a pooled conditional logit model.

### The Survey

The survey comprised 5 sections (see Supplementary Appendix 2): an introduction to the study and training materials, questions about respondents’ views on the information, the DCE questions, questions about respondents’ experience of completing the DCE questions, and demographic questions. Upon clicking the link to enter the survey, respondents were randomized to receive 1 of 2 versions of the online survey that used training materials presented as an animated storyline (“animation” version) or the text-based information (see Supplementary Appendix 1.1) as provided in the NBS leaflet in the United Kingdom (“leaflet” version). Both versions of the survey contained pages that summarized the attributes, their definitions, and levels using text-based information (see Appendix 1.1). This was necessary so that all respondents had the opportunity to go and re-read the definition of each attribute in the choice questions that were presented using summary headings for each attribute.

After reading the information or watching the animation, respondents were asked to rate their perceived degree of understanding on a 5-point scale (1 = *very easy* to 5 = *very hard*) and if they felt it would enable informed consent for NBS. After completing the 7 choice questions in the DCE, respondents were asked whether they believed they would make the same choices in real life and the degree of difficulty when making the choices in the survey. Respondents were also asked if they took account of all 4 attributes when making a choice.

In the final section of the survey, respondents were asked 2 attitudinal questions and 19 questions to understand their background and experiences. The first attitudinal question used to Health Information Orientation Scale to seek to determine the degree to which respondents felt they engage with and/or are apprehensive about receiving health-related information.^35,36^ The second attitudinal question asked respondents to rate the degree to which they agreed or disagreed with 6 ethical statements about NBS. The demographic questions included participants’ gender, number of previous children, religion, level of education, whether they or their partner were currently pregnant, and whether they had children who had previously received NBS.

### Training Materials

Two versions of training materials to be used at the start of the online survey were created: a text-based version using information from the current UK NBS leaflet and an animation. In the DCE, participants were randomized to receive the version containing only leaflet-based information or the version containing only the animation-based information. The NBS-leaflet version used screenshots of the relevant pages from the NBS section of the leaflet “Screening Tests for You and Your Baby.” In the United Kingdom, prior to May 2019, this leaflet was supposed to be given to women and their partners around weeks 8 to 12 during the pregnancy.^37^ The animation version was built around a linear storyline that aimed to convey the same information as the leaflet but also emphasized that the parents had a choice to make when taking part in NBS. The animation for use in the second arm of the DCE was developed by the research team as part of this study. The animation version (see Supplementary Appendix 1.2) was created by first writing a script that animators (SciAni)^38^ turned into a “cartoon” style story. Three key elements were factored in the design and how the individuals in the animation were portrayed: mix of ethnic backgrounds, gender-neutral colors for the baby’s clothes, and mix of family types. This creative process was iterative and involved interaction between the animators, the research team, and 3 experts (senior midwife, laboratory scientist, NBS program coordinator). The rationale and development of the script for the animation version was informed by a study published by Ulph et al. that that included qualitative interviews with 45 parents and 37 health professionals, an observation study with 8 midwives. In addition, the potential role of using non–text-based information strategies was explored in a DCE completed by 800 respondents including parents.^1,20,21^

### Pilot Study

A version of the online survey was pilot tested by 3 members of the public and qualitative feedback collated on the survey. Based on their feedback, only minor changes were made to the descriptions of the attributes and levels. Two quantitative pilot tests of the survey, each comprising 50 respondents, were then fielded to check the coding of the DCE and that there were no potential problems in analyzing the data. The 100 respondents were identified using the same sample frame as the main survey. No changes were made to the survey based on these quantitative pilot tests.

### Study Sample

The sample size required to yield statistically significant parameter estimates from the observed choice data is determined by a number of factors including the number of choice sets, profiles in each choice set, and levels on each attribute. In addition, the degree of preference heterogeneity in the responses also drives the required sample size.^39^ This study took a pragmatic approach to sample size calculation and aimed to recruit a sample of 1,000 members of an Internet panel (using the market research company Dynata).^40^ Respondents between the ages of 18 and 45 y were targeted to make the scenario of using a NBS program potentially relevant. The study sample was equally divided (250 in each group) into 4 target groups: men with children, women with children, men without children, and women without children.

### Data Collection

The online survey was programmed using Lighthouse Studio version 9.8.1^41^ and hosted on a secure server held by the University of Manchester. All data were held on this server. Potential respondents, recruited by Dynata, were sent a link to the survey. On completing the survey, the respondents were directed back to the Dynata Web site to receive reimbursement for taking part. The survey was fielded in February and March 2020 with data collection completed before the COVID-19 pandemic.

### Data Analysis

#### Choosing the logistic regression model specification

The first part of the analysis explored the choice data collected from the 2 versions of the online survey (animation or leaflet) using logistic regression models to understand respondents’ preferences for an NBS program and comprised 4 steps. The first step was the identification of the best specification of the regression model for the combined sample of respondents who had completed the animation and leaflet version of the online survey. A conditional logistic regression model was estimated with all the attributes coded assuming the movement between levels was linear and continuous. An alternative specific constant was included to represent the probability that participants would choose a screening service with no false-positive results, no wait for results, and mean effects for the storage of the bloodspot and treatment benefit compared with no screening. The robustness of this assumption about linearity was investigated using the approaches described in Supplementary Appendix 1.3. After choosing the best functional form of the model, uncorrelated and correlated random parameter models were then estimated to allow for potential preference heterogeneity and preference and scale heterogeneity in the data, respectively. The best model was chosen by using the Bayesian information criterion (BIC), which takes account of the impact of large number of parameters in a model and mitigates against the risk of overfitting.

#### Comparing preferences by mode of information delivery

The second part of the data analysis aimed to understand whether the animation version of the survey changed respondents’ ability to state their preferences for an NBS program and comprised 2 steps. The first step of this analysis aimed to determine the degree of error variance in the stated preferences from the observed choice data collected from each version of the online survey (animation or leaflet). The concept of “error variance” is used to refer to any influence or factor that people use to make their choices that is not explicitly described in the attributes and levels used in the experimental design. It was hypothesized that information that was more engaging would result in participants having a better understanding of the key attributes of a screening program, thereby exhibiting less error when expressing their preferences. Using the results of the best-fitting regression model estimated in step 4 of part 1 of the analysis, marginal rates of substitution (MRS) were calculated to control for potential scale heterogeneity as the result of comparing between 2 survey versions.^42,43^ Calculating MRS requires specification of an attribute to use in the denominator. Confidence intervals for the MRS were calculated using the delta method^44^, with statistical significance set at *P* = 0.05. If the estimated confidence intervals for the MRS of each attribute overlapped, then it was concluded that there was no difference in the preferences between the respondents completing the animation or leaflet version of the online survey (preference homogeneity). The second part of this analysis aimed to quantify the degree of error variance (testing for scale heterogeneity) in the estimated coefficients for each online version of the survey (animation or leaflet) using a heteroskedastic conditional logistic regression. The scale parameter, which was used to quantify the observed error variance in each group of respondents (text version or animation version) was estimated for the full sample of respondents and excluding those respondents who failed the dominance test question. In addition, the number of participants who always chose the profile with a better level (attribute dominance) of the time to results attribute or false-positive results attribute was calculated and the scale parameter estimated with and without these participants included in the data.

#### Impact of different modes of information delivery on sample subgroups

The next stage of the analysis aimed to determine whether a particular version of the online survey (animation or leaflet) had an impact on preferences for specified subgroups of the respondents. Individual heteroskedastic conditional logistic regression models were estimated for a prespecified list of subgroups (men v. women, people with previous children v. people with no previous children, different age bands, women or men whose partners were currently pregnant v. those who were not, and people who had previously received screening v. those who had not).

#### Effect of different modes of information delivery on self-reported understanding and ease of decision making

The final stage of the analysis aimed to identify whether the mode of information provision (animation or leaflet) influenced the degree of self-reported level of understanding by respondents completing each version of the online survey. Data from the 2 questions collected using the 5-point rating scale, summarized as a mean overall score for each question, were analyzed. The mean scores for the animation version and leaflet version were compared using Mann-Whitney *U* tests (with a statistical difference defined as *P* < 0.05). The mean scores for each prespecified demographic group were also compared to explore whether the leaflet or video were particularly well or poorly understood by different groups.

## Results

A total of 1,000 respondents (see Table 2) completed the online survey (animation version = 475 respondents; leaflet version = 525 respondents). Participants spent a median of 52 s reading the text-based information and 6.22 min watching the animation. Details of the demographic makeup of the 2 groups can be found in Table 2. Of the 1,000 respondents, 176 (17.6%) failed the internal validity test but were retained in the data set in line with common practice.^45^ The observed failure rate of 17.6% was less than the 30% predicted by the method proposed by Tervonen and colleagues, indicating that the data were of good quality.^34^ In total, 19.4% of the sample displayed dominant preferences for a single attribute, with most of these always choosing profiles with lower false-positive rates (15.1%). A minority of individuals (*n* = 16, 1.6%) always chose the no-screening program option in each choice set, and these were retained in the data set as it is possible that some respondents object to NBS on religious or other grounds. These respondents were divided equally between the animation-version (*n* = 8) and leaflet version of the survey (*n* = 8), suggesting that the mode of information provision did not influence respondents’ preferences about whether to opt-out of the NBS as described.

**Table 2:** Respondent Characteristics

Characteristic	Animation Version, *n* = 475 (%)	Leaflet Version, *n* = 525 (%)
Gender		
Male	236 (49.7)	264 (50.3)
Female	239 (50.3)	261 (49.7)
Age band, y		
18 to 24	55 (11.6)	65 (12.4)
25 to 34	178 (37.4)	189 (36.0)
35 to 45	242 (50.9)	271 (51.6)
Number of children		
None	233 (49.1)	267 (50.9)
1	130 (27.4)	113 (21.5)
2	78 (16.4)	108 (20.6)
≥3	34 (7.2)	37 (7.0)
Level of education		
No formal qualifications	7 (1.4)	4 (0.8)
1 to 4 O-levels/GCSEs	17 (3.8)	12 (2.3)
5+ O-levels/GCSEs	37 (7.8)	41 (7.8)
National vocational qualifications	45 (9.4)	47 (9.0)
A-levels/AS-levels	91 (19.2)	110 (21.0)
Undergraduate degree	173 (36.4)	204 (38.9)
Master’s degree	81 (17.1)	87 (16.6)
PhD	18 (3.8)	13 (2.5)
Other formal qualification	6 (1.3)	6 (1.1)
Religion		
No religion	248 (52.2)	270 (51.4)
Christian	178 (37.5)	191 (36.4)
Buddhist	10 (2.1)	9 (1.7)
Hindu	8 (1.6)	16 (3.0)
Jewish	1 (0.2)	6 (1.1)
Muslim	24 (5.1)	26 (5.0)
Sikh	2 (0.4)	2 (0.4)
Other	4 (0.8)	5 (1.0)
Currently pregnant (or partner is pregnant)		
Yes	32 (6.7)	38 (7.2)
No	436 (91.8)	480 (91.4)
Don’t know	7 (1.5)	6 (1.1)
Number of previous pregnancies		
None	237 (49.9)	263 (50.1)
1	116 (24.4)	111 (21.1)
2	82 (17.3)	87 (16.6)
≥3	40 (8.4)	63 (12.0)
Previously offered newborn bloodspot screening		
Yes	92 (19.4)	105 (20.0)
No	120 (25.2)	122 (23.2)
Don’t know	30 (6.3)	31 (5.9)

GCSE, general certificate of secondary education.

### Preferences for a NBS Program

The uncorrelated random parameter logit model had the lowest BIC of the 3 candidate models and was chosen to compare the preferences of the 2 groups. Table 3 shows the results of this model with the attributes coded as described in Table 1. These results indicated homogeneity in the preferences across the respondents completing each of the 2 versions (animation or leaflet) of the online survey. The calculated MRS are also shown using “time to results” as the denominator. Supplementary Appendix 1.3 shows the results of the visual inspection tests for linearity in the 2 attributes: time to results and percentage of false positive. Supplementary Appendix 1.3 also outlines the results of the process taken to choose the correct functional form for these 2 attributes. The final chosen model used piecewise parameters for false-positive rates with a knot at 5% to allow for nonlinear preferences.^46^

**Table 3:** Results of the Uncorrelated Random Parameter Logit Model to Estimate Preferences for a Newborn Bloodspot Screening Program in the United Kingdom

Attribute and Level	Video			Leaflet		
	Coefficient	MRS	CIs	Coefficient	MRS	CIs
Constant for NBS compared with no screening	7.180***	210.68	154.69 to 266.67	5.994***	194.27	146.36 to 242.19
Treatment stops progression	0.685***	20.11	14.22 to 26.00	0.638***	20.66	14.86 to 26.47
Treatment slows progression	−0.159**	−4.66	−7.59 to −1.73	−0.092*	−2.98	−5.62 to −0.34
Treatment reduces symptoms	−0.527***	−15.45	−20.57 to 10.33	−0.546***	−17.68	−22.98 to −12.39
Bloodspot is stored	0.005	0.138	−1.51 to 1.79	0.009	0.28	−1.35 to 1.92
Bloodspot is not stored	−0.005	−0.138	−1.79 to 1.51	−0.009	−0.28	−1.92 to 1.35
False-positive rate up to 5%	−0.230***	−6.74	−8.95 to −4.53	−0.2067***	−6.70	−8.90 to −4.50
False-positive rate more than 5%	−0.084**	−2.47	−4.24 to −0.70	−0.087***	−2.82	−4.53 to −1.09
Time to results	−0.034***	—	—	−0.031***		

CI, confidence interval; MRS, marginal rates of substitution; NBS, newborn bloodspot screening.

* Significant at the 5% level.

** Significant at the 1% level.

*** Significant at the 0.1% level.

A comparison of the calculated MRS (see Table 3), which corrects for the potential effect of scale heterogeneity,^42,43^ indicates that the preferences of respondents completing the animation version of the online survey did not statistically significantly differ from the observed preferences of respondents completing the leaflet version of the online survey. The observed result was less negative MRS for the attribute “time to result” for respondents who completed the leaflet version of the online survey, suggesting that this group showed a modest (albeit not statistically significant) preference for improvements in time to result as part of an NBS program.

The preferences from all respondents indicated they placed a high intrinsic value on participating in an NBS program. All respondents showed preferences in line with a priori expectations and disliked increasing levels of false-positive results in an NBS program. All respondents preferred an NBS program that featured conditions in which progression could be stopped by early treatment with a lower preference for an NBS program that only slowed disease progression or relieved symptoms from the conditions. None of the respondents indicated strong preferences for whether the bloodspot sample was stored. On average, respondents were willing to wait 129 d for a result (animation version) or 124 d for a result (leaflet version) as part of an NBS program. The preferences for attributes of an NBS in each group were not affected when participants who failed the internal validity test were removed.

### Impact of Mode of Information Provision on Stated Preferences for an NBS Program

Table 4 shows the results of a heteroskedastic conditional logistic regression to identify observed differences in the error variance of respondents completing each version (animation or leaflet) of the online survey. These results indicated that respondents who completed the animation version of the online survey exhibited less error variance in their responses. This result was statistically significant at the 5% level. The calculated scale parameter for the sample receiving the animation-based information in this analysis was 1.09 (95% confidence intervals: 1.01–1.18), indicating that those receiving the animation exhibited 9% less error variance in expressing their preferences. This scale parameter figure is calculated by taking the exponent of the error variance term produced in the heteroskedastic condition logit (0.088). When respondents who failed the dominance test were excluded from the sample, the estimated scale parameter for respondents who completed the survey with the animation version was 1.08 (95% confidence interval: 1.00–1.17). When respondents who always chose the better or worse levels of the continuous attributes were removed, the estimated scale parameter for participants completing the animation version was 1.09 (95% confidence interval 1.00–1.19). When both participants who failed the dominance test and participants with attribute-dominant preferences were removed, the estimated scale parameter rose to 1.10 (95% confidence interval 1.01–1.20).

**Table 4:** Results of the Heteroskedastic Conditional Logistic Model to Quantify Differences in Error Variance between the Samples

Attribute/Level	Coefficient	*P* Value	Confidence Interval
Constant for NBS compared with no screening	2.657***	0.001	2.499 to 2.814
Treatment stops progression	0.383***	0.001	0.336 to 0.429
Treatment slows progression	−0.079***	0.001	−0.121 to −0.037
Treatment reduces symptoms	−0.304***	0.001	−0.349 to −0.258
Bloodspot is stored	0.004	0.79	−0.023 to 0.030
Bloodspot is not stored	−0.004	0.79	−0.030 to 0.023
False-positive rate up to 5%	−0.137***	0.001	−0.163 to −0.112
False-positive rate over 5%	−0.041**	0.001	−0.065 to −0.018
Time to results	−0.021***	0.001	−0.025 to −0.017
Error variance term			
Video	0.088*	0.027	0.010 to 0.166

NBS, newborn bloodspot screening.*Significant at the 5% level.**Significant at the 1% level.***Significant at the 0.01% level.

Supplementary Appendix 1.4 reports the estimated scale parameter for subgroups of the respondent sample that completed the animation version of the online survey. This analysis suggested that using an animation was particularly effective at improving the error variance of choices (reducing the error variance) of respondents who were female, have children, and are aged between 35 and 45 y. The influence of the animation version for respondents with different levels of education was less clear. The error variance of the responses from people with degree-level education was improved by the animation version. However, although the animation improved the error variance of the responses from people educated to the level of secondary school exams, the observed error variance of the responses from people with vocational qualifications was higher. The sample size for these groups was small (17 and 45, respectively), so caution should be taken in interpreting the results. No significant effect of the animation version was observed in any religious group apart from Christians, who had lower error variance. However, this group, along with people with no religion, may have been the only groups with sufficient sample size to observe an effect.

### Self-Reported Level of Understanding

**Figure 2:**
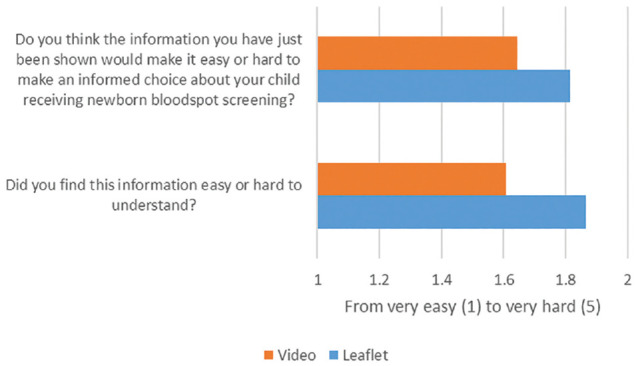
Self-reported level of understanding of training materials. 1. Mann-Whitney *U* test Prob > |z| = 0.008. 2. Mann-Whitney *U* test Prob > |z| = 0.000.

Figure 2 shows the results from the 2 questions that asked respondents to self-report the level of understanding once they had watched or read the training materials. These results show that respondents, in general, reported they found the information either “very easy” or “easy” to understand in both formats of the training materials (animation or leaflet). The Mann-Whitney *U* tests for statistically significant differences using the mean score from these questions indicate that, on average, respondents watching the animation self-reported that it was easier to provide informed consent based on this information. Removing participants who had failed the internal validity test did not affect the mean self-reported understanding or ability to provide informed consent of either group.

Self-reported ease of understanding of the information was better for the video compared with the leaflet across demographic groups, with the exception of individuals with 5+ O levels or general certificates of secondary education, national vocational qualifications (NVQ), and Buddhist or Sikh religion (see Supplementary Appendix SA1.4.2). While these groups had a small sample size, the finding that the video was worse for participants with NVQs (although not statistically significantly) aligns with the finding from the DCE that the video resulted in higher error variance for these participants.

## Discussion and Conclusions

### Discussion

This study was motivated by the premise that animation-based training materials, rather than the standard text-based leaflet format, would facilitate information provision for respondents indicating their preferences for a NBS program.^1^ It was hypothesized that if an animation was used, respondents’ preferences for a NBS program would not be influenced but that there would be more less error variance in the observed stated preferences. This study, using an age-stratified representative sample of the UK public, suggested that using an animation as training materials did not change the observed stated preferences for a NBS program, but it did change the error variance of the stated preferences. The reduced error variance of preferences was particularly relevant in respondents who were female, with children, between the ages of 35 and 45 y, and with a degree-level education. However, the video increased error variance for individuals with NVQs. More participants in the video arm reported that the information was easy to understand as compared with those in the leaflet arm, and this finding was generally consistent across demographic groups, with the exception of some lower education level groups. The results of this study suggest that introducing an animated storyline into the package of information currently provided to inform parents about bloodspot screening as part of a national program may be beneficial, although individuals should be able to choose which mode of information provision is most suitable to them.

This study indicated all participating respondents would take up an NBS program as described in the choice experiment in instances in which the NBS program could enable the early detection of conditions whose progression could be stopped by early treatment. In contrast, respondents were less likely to choose an NBS program in which early detection only slowed the disease or relieved symptoms. These finding have face validity suggesting that respondents were engaging with the survey questions. Neither the storage of the bloodspot sample nor increased rates of false-positive results were strong drivers of preferences for an NBS program in this study.

There is some evidence that using animations for information provision may provide more effective information exchange than traditional text-based information. For example, 1 DCE compared the effect of information provided in text or via an animated storyline on the preferences of members of the public for a biologic calculator in rheumatoid arthritis.^47^ The researchers found evidence that respondents receiving an animated storyline as part of the DCE training materials exhibited 20% less error variance in their responses than those receiving text-based information. In another DCE and broader survey to investigate the effects of video versus text-based information on preferences for ovarian cancer treatment, no differences in error variance were identified, but respondents receiving the video were more likely to correctly answer questions testing respondents’ understanding of the information.^48^ These results suggest that including engaging training materials may be beneficial in producing results with less error variance, and therefore greater statistical significance, from the same sample of participants. Researchers should also be aware that the design and conduct of a DCE is an art not a science that can follow a formulaic approach. Producing animation-style training materials is more expensive than generating text versions. Therefore, researchers need to assess whether the benefits (more informative results) are worth the additional costs when compared with other strategies such as increasing the sample size completing the survey. To address this question would require a formal evaluation using a cost-effectiveness framework to quantify the incremental costs and benefits of animation versions of training materials compared with expanding the sample frame.^49^

The key limitation of this study is the use of responses to a DCE as a proxy for parents’ level of understanding of NBS for the purpose of providing informed consent for screening. To reduce the complexity of the choice tasks, only 4 attributes were used, and in reality, parents may base the decision as to whether their child should be screened on more aspects of NBS. It is therefore important that the video-based animated storyline is fully tested to determine whether the improvements in error variance identified in this study are reflected in improvements in the ability of parents to provide informed consent for screening. This is particularly important given potential indications that the animation may have increased error variance for participants with vocational qualifications. Further qualitative research may help to explain why the animation appeared to increase error variance for this group while reducing error variance for women, people with children, and people between the ages of 35 and 45 y. When comparing these with results with the self-reported ease of understanding of the information, the groups that exhibited lower error variance in the video arm also appeared to have a high reported level of understanding across both the leaflet and video arms. It may be that those with previous children remember receiving previous information about NBS and that people between the ages of 35 and 45 y are more likely to have children in the sample. In addition, women who have had children may be more likely to be the primary decision maker about a child’s health, meaning that they have engaged more with previous information and the information presented in the DCE. Further qualitative research would be needed to explore these hypotheses.

When directly asked how easy it would be to provide informed consent based on the information provided in the animated storyline, respondents provided an average rating that lay between “very easy” and “easy,” and it was deemed easier to provide informed consent based on the animated storyline than text-based information. However, there may be other aspects of the delivery of the video and leaflet that affect people’s ability to access and take on board the information provided. For example, parents would have to take the time to find a video on the internet on their computer or smartphone. Some parents may not have easy access to the internet, and so a focus on video-based information may not be an equitable solution to informing parents about NBS. Participants also spent much longer watching the animation than reading the leaflet. This may be because the video is more engaging or may indicate that participants did not pay sufficient attention to the leaflet-based information. Further research is needed to determine how long it takes to read the information in full in order to check whether the reading times in this study were too fast.

While the animation was designed using the same content of information as the leaflets, there were some differences between the versions. The animation contained orientating statements at the beginning including the reasons why patients were receiving the information and why screening was conducted, which were not present at the beginning of the leaflet-based information. The animation specifically mentions midwives as giving the information, whereas the leaflet refers more generically to health professionals. The animation directly states that individuals can choose to screen or not screen, while the leaflet does not specifically mention the ability to fully opt out. Finally, the animation mentions that there is a choice as to whether the blood spot is stored, whereas the leaflet states that it is stored for 5 y. Other aspects of the video may have driven differences in preferences, and it is not possible to fully disentangle these. For example, the animation was designed using diverse ethnicities in health professional roles, whereas the leaflet features very few images in general. This diversity, among other varying factors, may hypothetically have increased engagement with the materials beyond the effect of video versus leaflet-based information.

Combined, the differences between the versions of information shown to participants make it very difficult to disentangle which aspect of the animation made it easier to understand and resulted in lower error variance than the text-based information. Further qualitative research would be beneficial to identify which specific aspects of the information were beneficial from both a clinical and methodological perspective. However, an interesting finding of this study is that, despite these differences, particularly with regard to the presentation of choice about screening and bloodspot storage, there was no difference in preferences for the screening program itself.

## Conclusion

This result supporting the use of animation to provide information is of relevance for analysts designing training materials as part of choice experiments to elicit preferences for health care interventions. This finding is also relevant to inform the provision of different formats of information to enable informed choice in the health care context as part of national blood spot screening programs. This study also demonstrated that different modes of information provision may have different impacts on participants’ ability to express their preferences. As such, service providers should consider providing informational materials in a variety of formats so that patients can choose the mode of information delivery they find most engaging.

## Supplemental Material

sj-docx-1-mpp-10.1177_23814683241232935 – Supplemental material for Understanding the Impact of Different Modes of Information Provision on Preferences for a Newborn Bloodspot Screening Program in the United Kingdom Supplemental material, sj-docx-1-mpp-10.1177_23814683241232935 for Understanding the Impact of Different Modes of Information Provision on Preferences for a Newborn Bloodspot Screening Program in the United Kingdom by Stuart J. Wright, Caroline M. Vass, Fiona Ulph and Katherine Payne in MDM Policy & Practice

sj-pdf-1-mpp-10.1177_23814683241232935 – Supplemental material for Understanding the Impact of Different Modes of Information Provision on Preferences for a Newborn Bloodspot Screening Program in the United Kingdom Supplemental material, sj-pdf-1-mpp-10.1177_23814683241232935 for Understanding the Impact of Different Modes of Information Provision on Preferences for a Newborn Bloodspot Screening Program in the United Kingdom by Stuart J. Wright, Caroline M. Vass, Fiona Ulph and Katherine Payne in MDM Policy & Practice

## References

[1] Ulph F Wright S Dharni N , et al. Provision of information about newborn screening antenatally: a sequential exploratory mixed-methods project. Health Technol Assess. 2017;21(55):1–240. 10.3310/hta21550 PMC5641821 28967862

[2] Public Health England. Newborn blood spot screening: programme overview. 2021. Available from: https://www.gov.uk/guidance/newborn-blood-spot-screening-programme-overview [Accessed 9 June, 2021].

[3] Nicholls SG Southern KW. Parental decision-making and acceptance of newborn bloodspot screening: an exploratory study. PLoS One. 2013;8(11). Available from: https://pubmed.ncbi.nlm.nih.gov/24265771/ [Accessed 9 June, 2021]. 10.1371/journal.pone.0079441 PMC3827133 24265771

[4] Hayeems RZ Miller FA Bombard Y , et al. Expectations and values about expanded newborn screening: a public engagement study. Health Expect. 2015;18(3):419–29. 10.1111/hex.12047 PMC5060787 23369110

[5] Tluczek A Orland KM Nick SW Brown RL. Newborn screening: an appeal for improved parent education. J Perinat Neonatal Nurs. 2009;23(4):326–34. 10.1097/JPN.0b013e3181a1bc1f PMC2947955 19915416

[6] Public Health England. Newborn blood spot screening: programme handbook. 2018. Available from: https://www.gov.uk/government/publications/health-professional-handbook-newborn-blood-spot-screening [Accessed 9 June, 2021].

[7] Public Health England. Screening tests for you and your baby (STFYAYB). 2021. Available from: https://www.gov.uk/government/publications/screening-tests-for-you-and-your-baby [Accessed 9 June, 2021].

[8] National Health Service. Newborn blood spot test. 2021. Available from: https://www.nhs.uk/conditions/baby/newborn-screening/blood-spot-test/ [Accessed 25 October, 2021].

[9] National Health Service. Screening tests in pregnancy. 2021. Available from: https://www.nhs.uk/pregnancy/your-pregnancy-care/screening-tests/ [Accessed 25 October, 2021].

[10] Davey A French D Dawkins H O’Leary P. New mothers’ awareness of newborn screening, and their attitudes to the retention and use of screening samples for research purposes. Genomics Soc Policy. 2005;1(3). Available from: https://www.ncbi.nlm.nih.gov/pmc/articles/PMC5424959/ [Accessed 4 October, 2021].

[11] Kerruish N Webster D Dickson N. Information and consent for newborn screening: practices and attitudes of service providers. J Med Ethics. 2008;34(9):648–52. 10.1136/jme.2007.023374 18757632

[12] Moody L Choudhry K. Parental views on informed consent for expanded newborn screening. Health Expect. 2013;16(3):239–50. 10.1111/j.1369-7625.2011.00710.x PMC5060664 21838829

[13] Fant K Clark S Kemper A. Completeness and complexity of information available to parents from newborn-screening programs. Pediatrics. 2005;115(5):1268–72. 10.1542/peds.2004-0834 15867034

[14] Hargreaves KM Stewart RJ Oliver SR. Informed choice and public health screening for children: the case of blood spot screening. Health Expect. 2005;8(2):161–71. 10.1111/j.1369-7625.2005.00324.x PMC5060287 15860056

[15] Nicholls SG Southern KW. Parental information use in the context of newborn bloodspot screening. An exploratory mixed methods study. J Community Genet. 2012;3(4):251–7. 10.1007/s12687-012-0082-4 PMC3461221 22350979

[16] Detmar S Hosli E Dijkstra N Nijsingh N Rijnders M Verweij M. Information and informed consent for neonatal screening: opinions and preferences of parents. Birth. 2007;34(3):238–44. 10.1111/j.1523-536X.2007.00176.x 17718874

[17] Newcomb P True B Walsh J Dyson M Lockwood S Douglas B. Maternal attitudes and knowledge about newborn screening. MCN Am J Matern Child Nurs. 2013;38(5):289–94. 10.1097/NMC.0b013e31829a55e2 23799342

[18] Ulph F Cullinan T Qureshi N Kai J. Parents’ responses to receiving sickle cell or cystic fibrosis carrier results for their child following newborn screening. Eur J Hum Genet. 2015;23(4):459. 25005733 10.1038/ejhg.2014.126 PMC4666569

[19] Yang YM Andrews S Peterson R Shah A Cepeda M. Prenatal sickle cell screening education effect on the follow-up rates of infants with sickle cell trait. Patient Educ Couns. 2000;39(2–3):185–9. 10.1016/s0738-3991(99)00022-1 11040718

[20] Wright SJ Ulph F Lavender T Dharni N Payne K. Understanding midwives’ preferences for providing information about newborn bloodspot screening. MDM Policy Pract. 2018;3(1):238146831774617. 10.1177/2381468317746170 PMC6125045 30288434

[21] Wright SJ Ulph F Dharni N Payne K. Eliciting preferences for information provision in newborn bloodspot screening programs. Value Health. 2017;20(4):651–61. 10.1016/j.jval.2016.11.007 28408008

[22] Miller FA Hayeems RZ Bombard Y , et al. Public perceptions of the benefits and risks of newborn screening. Pediatrics. 2015;136(2):e413–23. 10.1542/peds.2015-0518 26169426

[23] Hendrix KS Meslin EM Carroll AE Downs SM. Attitudes about the use of newborn dried blood spots for research: a survey of underrepresented parents. Acad Pediatr. 2013;13(5):451–7. 10.1016/j.acap.2013.04.010 PMC4785598 24011748

[24] Soekhai V de Bekker-Grob EW Ellis AR Vass CM. Discrete choice experiments in health economics: past, present and future. Pharmacoeconomics. 2019;37(2):201–26. 10.1007/s40273-018-0734-2 PMC6386055 30392040

[25] Lancsar E Louviere J. Conducting discrete choice experiments to inform healthcare decision making: a user’s guide. Pharmacoeconomics. 2008;26(8):661–77. 10.2165/00019053-200826080-00004 18620460

[26] Bridges JF Hauber AB Marshall D , et al. Conjoint analysis applications in health—a checklist: a report of the ISPOR Good Research Practices for Conjoint Analysis Task Force. Value Health. 2011;14(4):403–13. 10.1016/j.jval.2010.11.013 21669364

[27] Ryan M Gerard K Amaya-Amaya M. Using discrete choice experiments to value health and health care. In: Ryan M Gerard K Amaya-Amaya M , eds. The Economics of Non-market Goods and Resources. Vol. 11. Dordrecht: Springer Netherlands; 2008. Available from: http://link.springer.com/10.1007/978-1-4020-5753-3 [Accessed 4 October, 2021].

[28] Vass CM Georgsson S Ulph F Payne K. Preferences for aspects of antenatal and newborn screening: a systematic review. BMC Pregnancy Childbirth. 2019;19(1):131. 30991967 10.1186/s12884-019-2278-7 PMC6469127

[29] Tarini BA Simon NJ Payne K Gebremariam A Rose A Prosser LA. An assessment of public preferences for newborn screening using best–worst scaling. J Pediatr. 2018;201:62–68.e1. 10.1016/j.jpeds.2018.05.035 30025667

[30] Bech M Kjaer T Lauridsen J. Does the number of choice sets matter? Results from a web survey applying a discrete choice experiment. Health Econ. 2011;20(3):273–86. 10.1002/hec.1587 20143304

[31] Kinter ET Prior TJ Carswell CI Bridges JFP . A comparison of two experimental design approaches in applying conjoint analysis in patient-centered outcomes research: a randomized trial. Patient. 2012;5(4):279–94. 10.1007/BF03262499 23145548

[32] Choicemetrics. NGene. 2019. Available from: http://www.choice-metrics.com/index.html

[33] Johnson F Lancsar E Marshall D , et al. Constructing experimental designs for discrete-choice experiments: report of the ISPOR conjoint analysis experimental design good research practices task. Value Health. 2013;16:3–13. 23337210 10.1016/j.jval.2012.08.2223

[34] Tervonen T Schmidt-Ott T Marsh K Bridges JFP Quaife M Janssen E. Assessing rationality in discrete choice experiments in health: an investigation into the use of dominance tests. Value Health. 2018;21(10):1192–7. 10.1016/j.jval.2018.04.1822 30314620

[35] Dubenske LL Burke Beckjord E Hawkins RP Gustafson DH. Psychometric evaluation of the health information orientation scale: a brief measure for assessing health information engagement and apprehension. J Health Psychol. 2009;14(6):721–30. 10.1177/1359105309338892 PMC2729509 19687109

[36] Bernard LL. Needs of Familial Caregivers of Cancer Patients across the Advanced Cancer Disease Trajectory. Denton: University of North Texas; 2004.

[37] Public Health England. Blood spot. 2021. Available from: https://www.gov.uk/government/publications/screening-tests-for-you-and-your-baby/blood-spot [Accessed 5 March, 2021].

[38] SciAni. Sci Ani – Science Animated – Your research brought to life. 2020. Available from: https://sciani.com/ [Accessed 12 June, 2020].

[39] de Bekker-Grob EW Donkers B Jonker MF Stolk EA. Sample size requirements for discrete-choice experiments in healthcare: a practical guide. Patient. 2015;8(5):373–84. 10.1007/s40271-015-0118-z PMC4575371 25726010

[40] Dynata. Dynata. 2021. Available from: https://www.dynata.com/ [Accessed 25 October, 2021].

[41] Sawtooth Software. Lighthouse Studio. Provo (UT): Sawtooth Software; 2020.

[42] Vass CM Wright S Burton M Payne K. Scale heterogeneity in healthcare discrete choice experiments: a primer. Patient. 2017; Available from: https://link.springer.com/article/10.1007%2Fs40271-017-0282-4 10.1007/s40271-017-0282-4 29032437

[43] Wright SJ Vass CM Sim G Burton M Fiebig DG Payne K. Accounting for scale heterogeneity in healthcare-related discrete choice experiments when comparing stated preferences: a systematic review. Patient. 2018;11(5):475–88. 10.1007/s40271-018-0304-x 29492903

[44] Hole AR. A Comparison of Approaches to Estimating Confidence Intervals for Willingness to Pay Measures. CHE Research Paper. York (UK): Centre for Health Economics; 2006. 10.1002/hec.1197 17238222

[45] Lancsar E Louviere J. Deleting ‘irrational’ responses from discrete choice experiments: a case of investigating or imposing preferences? Health Econ. 2006;15(8):797–811. 16615039 10.1002/hec.1104

[46] Van Der Pol M Currie G Kromm S Ryan M . Specification of the utility function in discrete choice experiments. Value Health. 2014;17(2):297–301. 24636390 10.1016/j.jval.2013.11.009

[47] Vass CM Davison NJ Vander Stichele G Payne K . A picture is worth a thousand words: the role of survey training materials in stated-preference studies. Patient. 13(2):163–73. 10.1007/s40271-019-00391-w PMC7075825 31565784

[48] Lim SL Yang JC Ehrisman J Havrilesky LJ Reed SD. Are videos or text better for describing attributes in stated-preference surveys? 2020;13(4). Available from: http://link.springer.com/10.1007/s40271-020-00416-9 [Accessed 6 April, 2020]. 10.1007/s40271-020-00416-9 32239442

[49] Drummond MF Sculpher MJ Torrance GW O’Brien BJ Stoddart GL. Methods for the Economic Evaluation of Health Care Programmes. Oxford: Oxford Medical Publications; 2005.

