# Supplementary material for: Understanding the Impact of Different Modes of Information Provision on Preferences for a Newborn Bloodspot Screening Program in the United Kingdom
Source: MDM Policy Pract. 2024 Mar 4;9(1):23814683241232935. doi: 10.1177/23814683241232935 (PMC10913504; doi:10.1177/23814683241232935)
Supplement: sj-docx-1-mpp-10.1177_23814683241232935 – Supplemental material for Understanding the Impact of Different Modes of Information Provision on Preferences for a Newborn Bloodspot Screening Program in the United Kingdom [file sj-docx-1-mpp-10.1177_23814683241232935.docx]

**Supplementary Appendix: Understanding the impact of different modes of information provision on preferences for a Newborn Bloodspot Screening Programme in the UK**

**Supplementary appendix**

**Supplementary Appendix 1.1:** The section of the current screening leaflet explaining Newborn Bloodspot Screening to parents living in the UK

**Supplementary Appendix 1.2:** Link to animation

**Supplementary Appendix 1.3:** Identification of non-linearity in continuous attributes

**Supplementary Appendix 1.4:** Estimated scale parameter for the animation version of the online survey

**Supplementary Appendix 1.1:** The section of the current screening leaflet explaining Newborn Bloodspot Screening to parents living in the UK


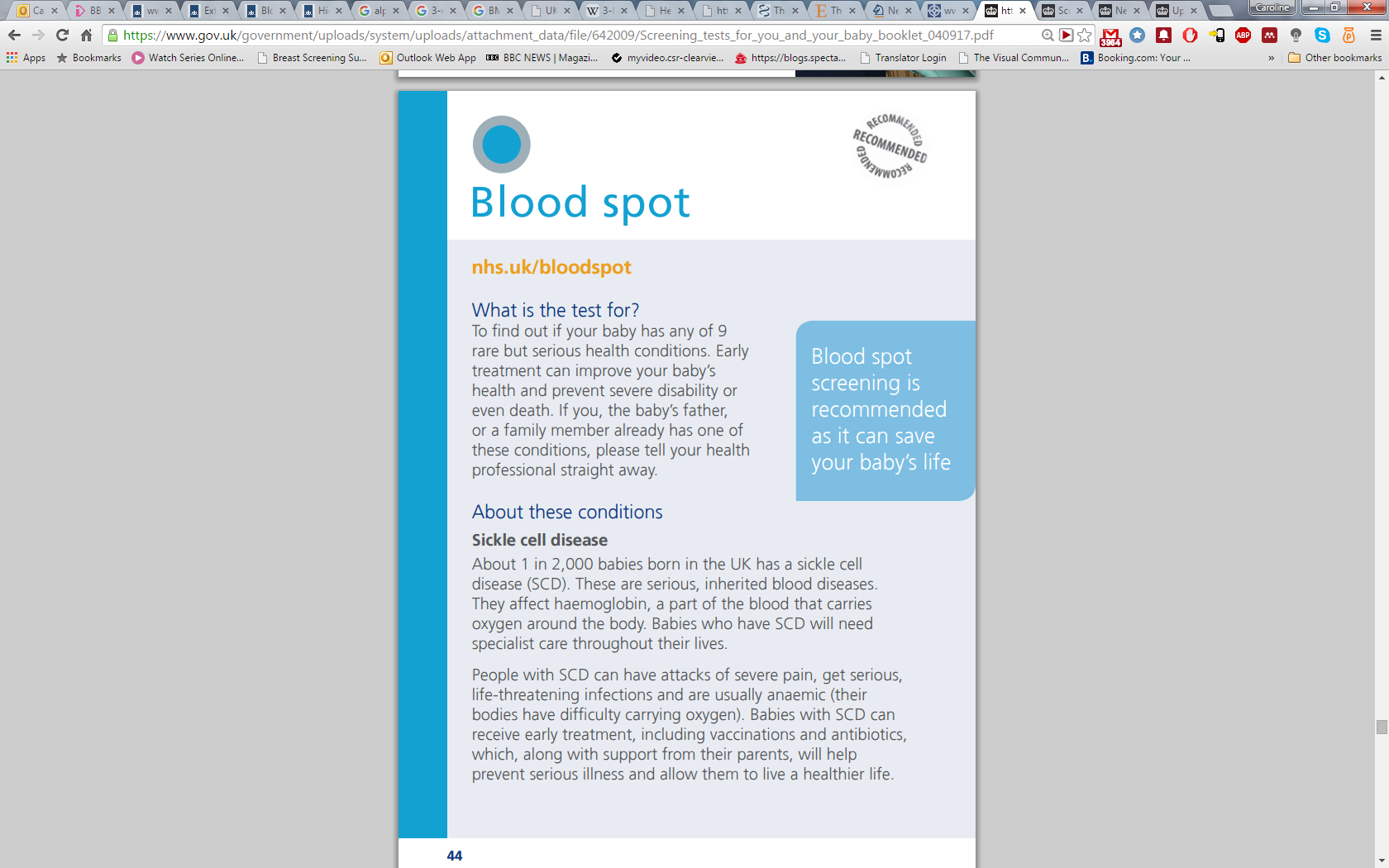


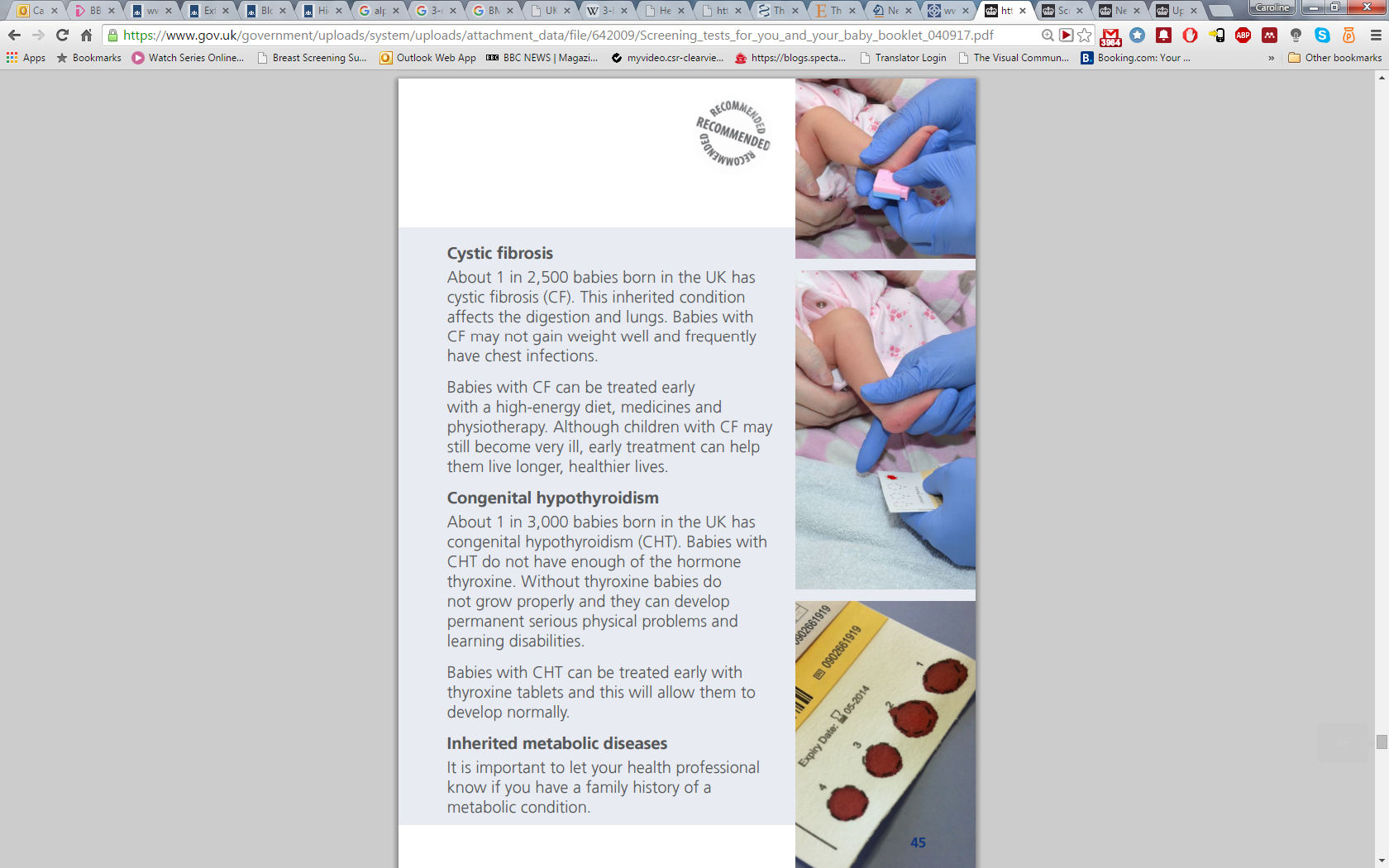


**
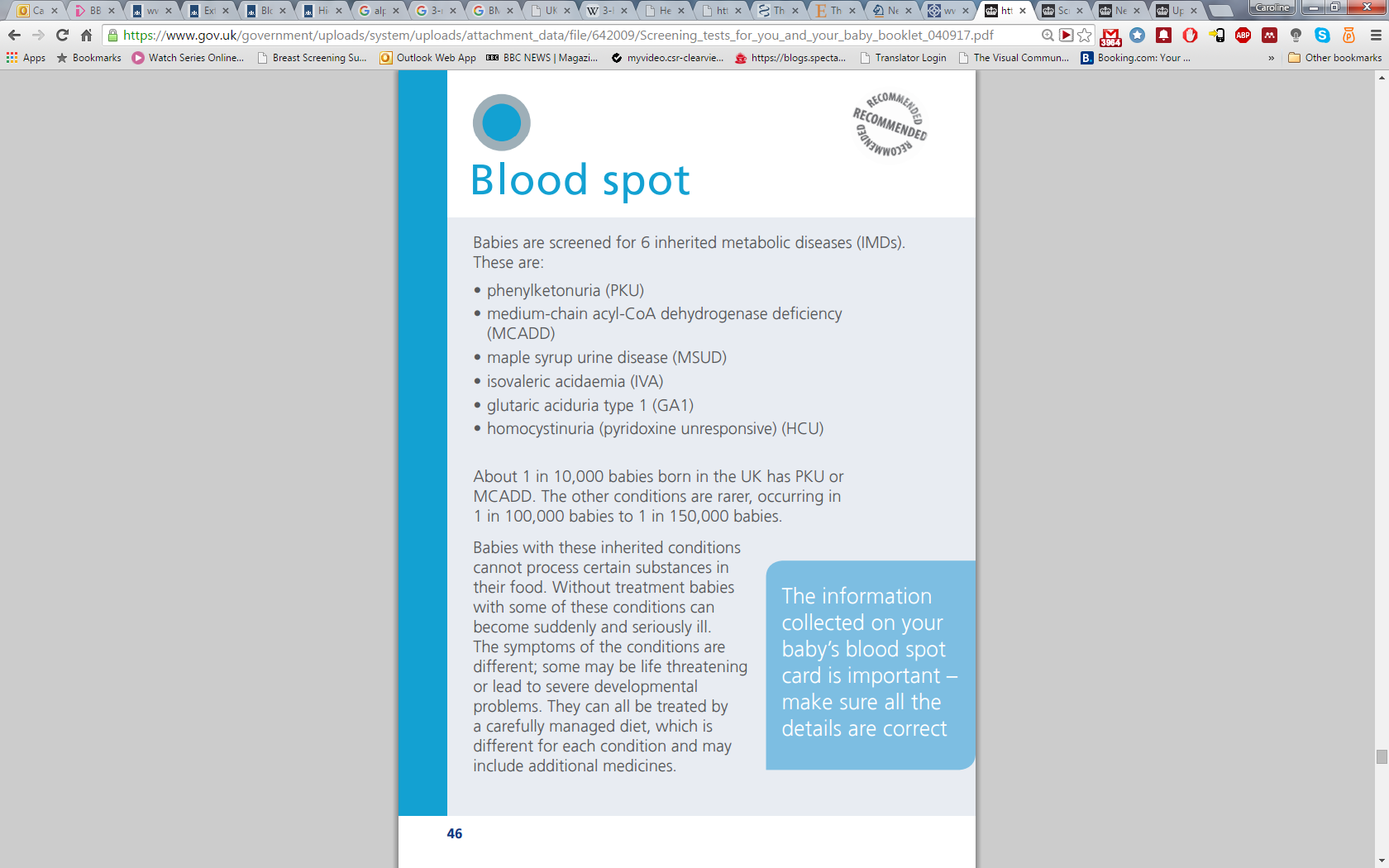
**

**
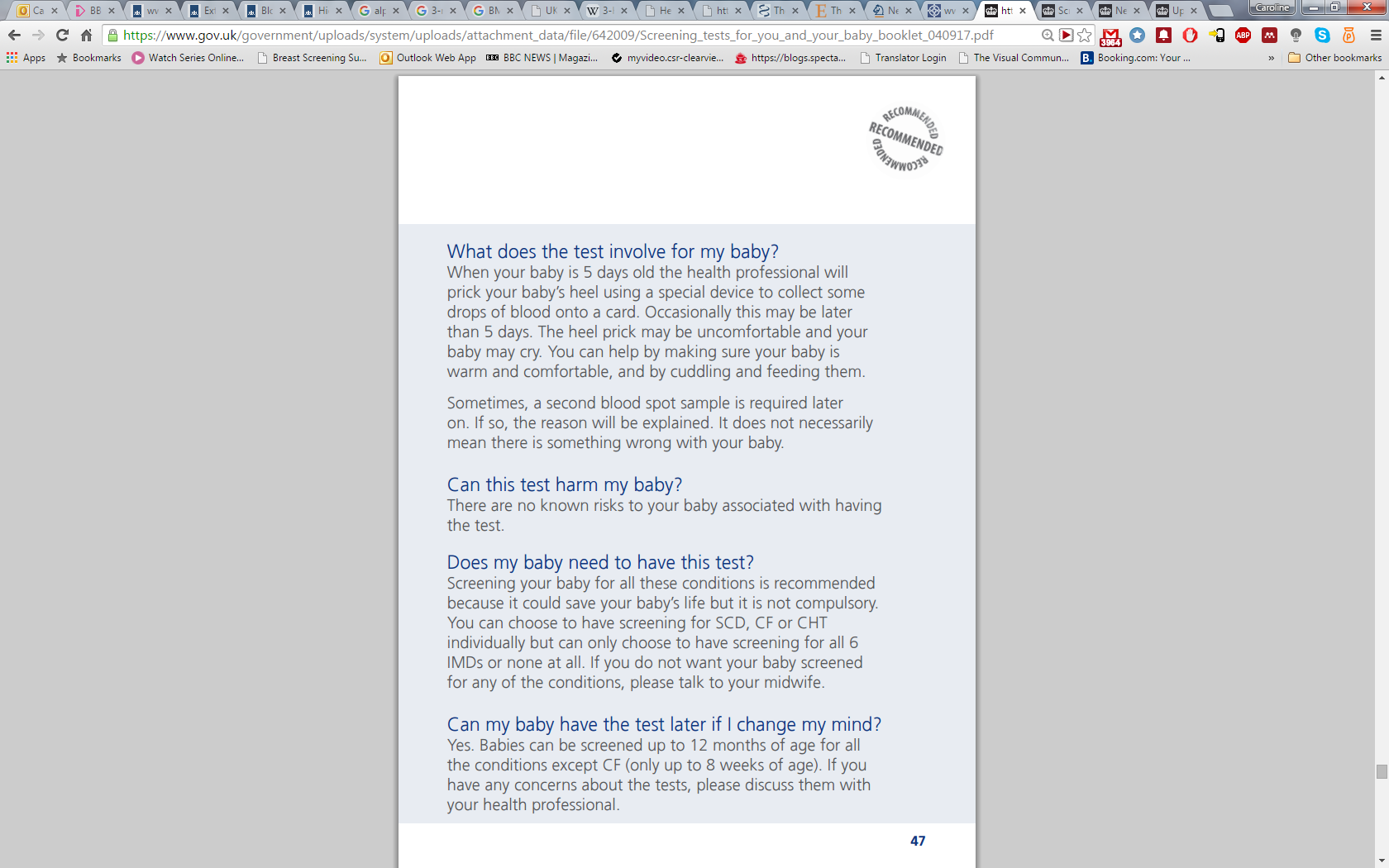
**

**
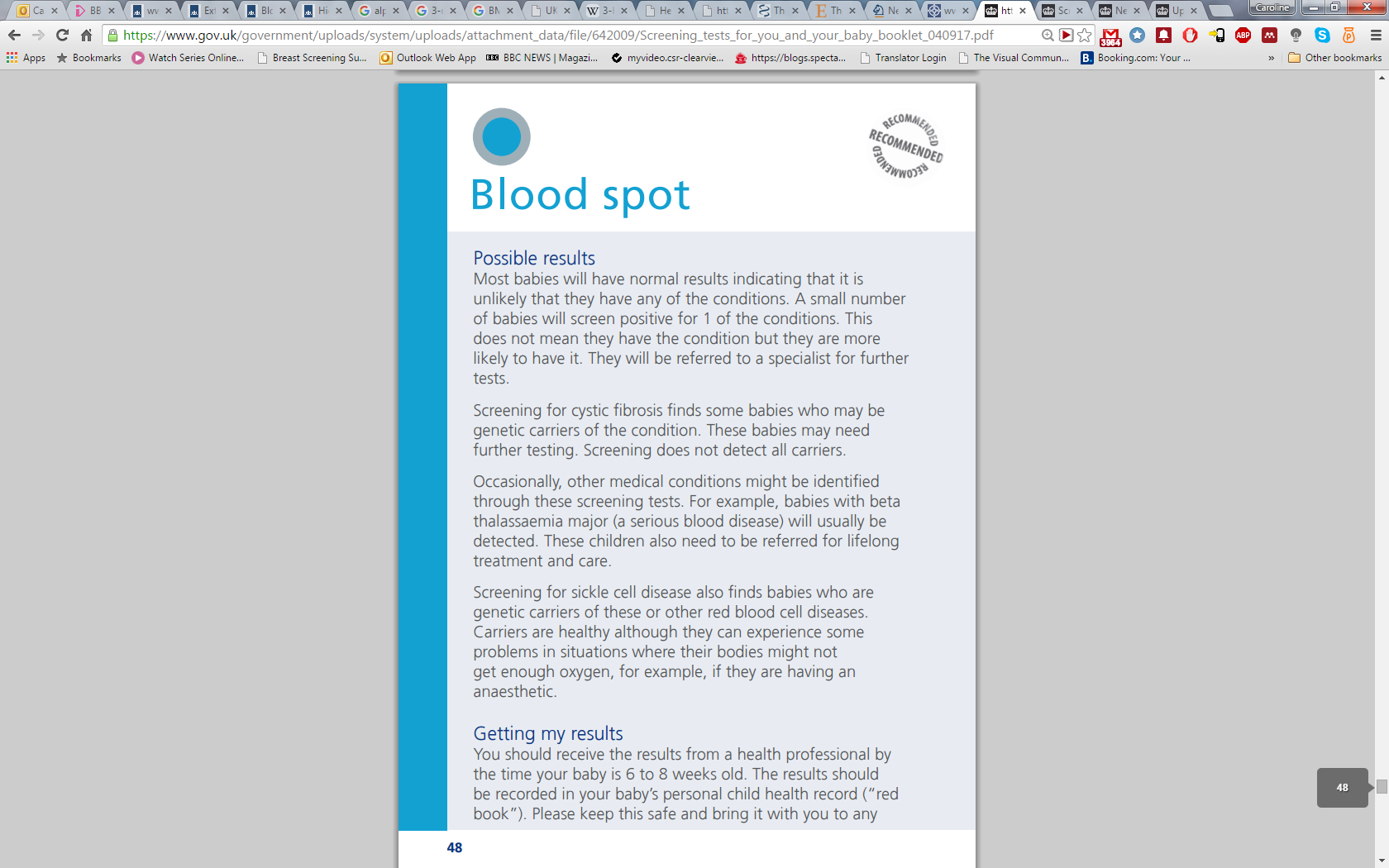
**

**
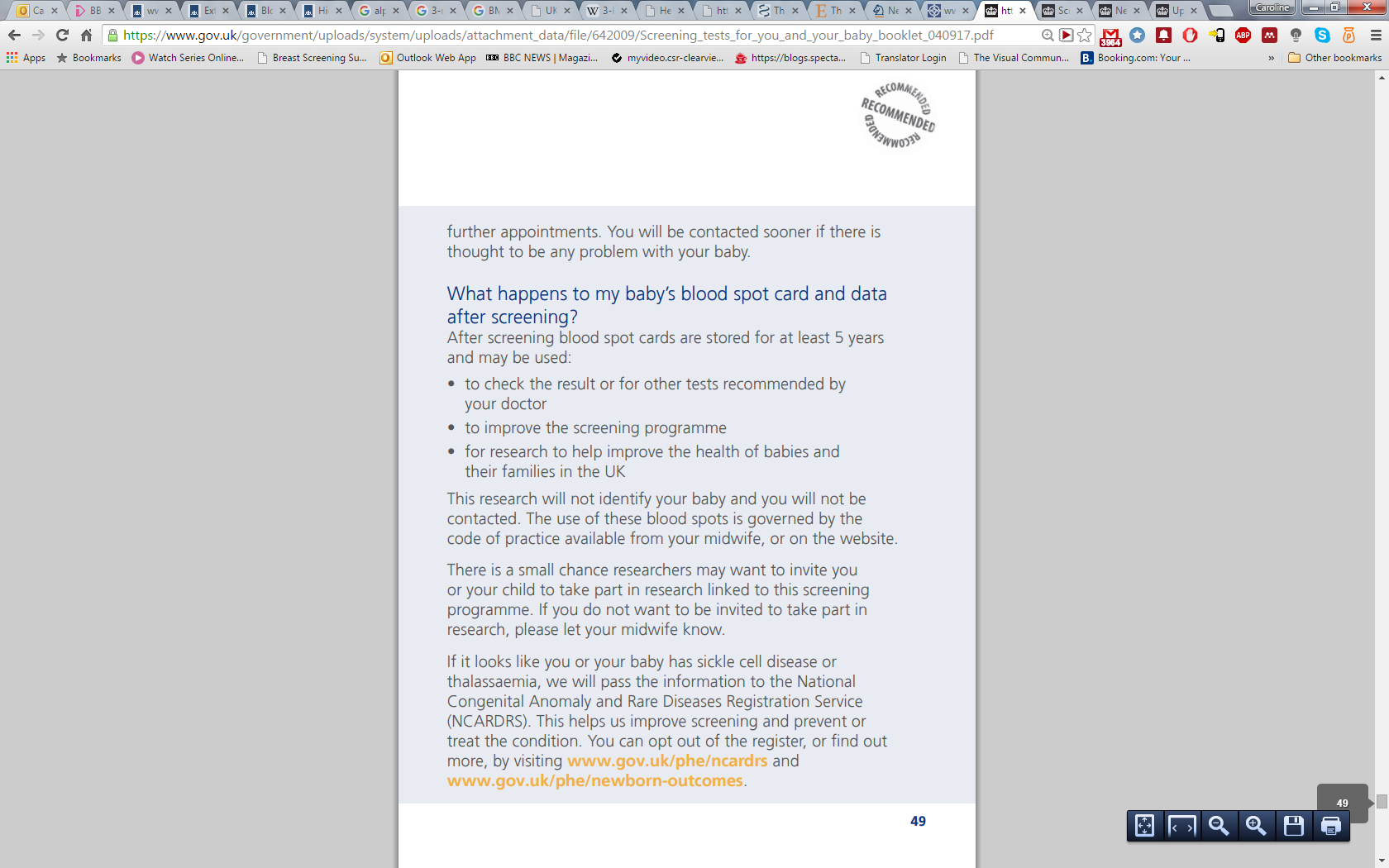
**

**Supplementary Appendix 1.2:** Link to animation

[**https://zenodo.org/records/8362707**](https://zenodo.org/records/8362707)

**Supplementary Appendix 1.3:** Identification of non-linearity in continuous attributes

This appendix shows the results of the visual inspection tests for linearity in the two potential attributes (time to results and percentage of false positive) and the approach to test for the appropriate specification of the functional form for the utility function.

Time to results

Figure SA1.3.1: Observation of the linearity of the time to result attribute


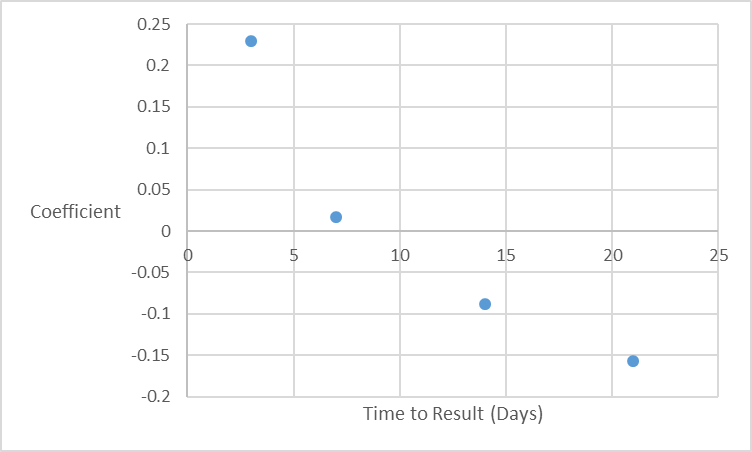


When plotted as an effects-coded variable, increasing times to receive the results of the test appeared to have a diminishing impact on the probability of choosing a profile. The conditional logistic regression model was re-run with quadratic, piecewise and log functions to account for the potential non-linearity. Table SA4.1 shows the log-likelihood, AIC, and BIC of each of these models. Both the AIC and BIC for the quadratic and piecewise specifications suggested that the addition of these components did not add sufficient explanatory power to the model to justify their inclusion. The inclusion of a log term for cost very marginally improved AIC and BIC but given this very small improvement in the model the researchers decided to maintain the linear specification for time to results.

Table SA1.3.1: Measures of model performance for different non-linear specifications of the time to results attribute

|  | LL | AIC | BIC |
| --- | --- | --- | --- |
| Linear | -5642.26 | 11296.52 | 11344.24 |
| Quadratic | -5642.24 | 11298.48 | 11354.14 |
| Piecewise | -5641.32 | 11296.64 | 11352.3 |
| Log | -5641.68 | 11295.37 | 11343.08 |

False-positive rate:

Figure SA1.3.2: Observation of the linearity of the false-positive rate attribute


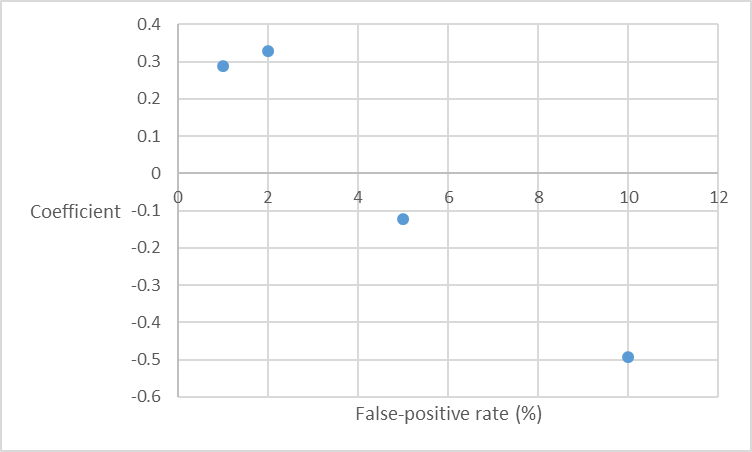


Observing the plotted effect-coded levels for the false-positive rate attribute, it can be seen that the 1 and 2% levels both had a similar effect on the probability of choosing a profile suggesting that respondents did not distinguish between the levels. Beyond this, there is some potential indication of a diminishing marginal effect of increasing false-positive rates on the probability of choosing a profile. Table SA1.3.2 shows the model performance measures for different specifications of non-linearity.

Table SA1.3..2: Measures of model performance for different non-linear specifications of the false-positive rate

|  | LL | AIC | BIC |
| --- | --- | --- | --- |
| Linear | -5642.26 | 11296.52 | 11344.24 |
| Quadratic | -5634.78 | 11283.57 | 11339.23 |
| Piecewise(5) | -5633.88 | 11281.77 | 11337.43 |
| Log | -5638.21 | 11288.42 | 11336.13 |

In this case, all of the different specifications of non-linearity improved the performance of the model. The best performing model based on the AIC is a piecewise specification of the false-positive rate with a break at the 5% level. On the other hand, the BIC suggests the log model is the best choice of model. This is likely because the piecewise model uses two parameters to specify the non-linearity and is therefore punished more in the calculation of BIC than the single parameter log model. Given that differences in the BIC between the model models are only very small, the piecewise specification was chosen for this analysis.

**Supplementary Appendix 1.4:** Estimated scale parameter for the animation version of the online survey

This analysis reports the estimated scale parameter calculated from the heteroscedastic regression model (see main text Table 5). The estimated scale parameter is calculated by taking the exponential of the coefficient for each attribute.

Table SA1.4.1: Estimated scale parameter for the animation version of the online survey

| **Variable** | **Scale parameter** | **95% confidence interval for the scale parameter** | **P value*** |
| --- | --- | --- | --- |
| Male | 1.007 | 0.897 to 1.129 | 0.911 |
| Female | 1.200*** | 1.077 to 1.338 | 0.001* |
| No previous children | 1.073 | 0.963 to 1.195 | 0.203 |
| Previous children | 1.165** | 1.039 to 1.307 | 0.009* |
| Age: 18 to 24 years | 1.173 | 0.919 to 1.496 | 0.200 |
| Age: 25 to 34 years | 1.087 | 0.951 to 1.242 | 0.224 |
| Age: 35 to 45 years | 1.118* | 1.005 to 1.243 | 0.040* |
| Currently pregnant | 0.867 | 0.560 to 1.343 | 0.524 |
| Previously had screening | 1.146 | 0.960 to 1.368 | 0.132 |
| No formal qualifications | 0.696 | 0.343 to 1.411 | 0.314 |
| 1 to 4 O-levels/GCSEs | 2.520** | 1.349 to 4.707 | 0.004 |
| 5+ O-levels/GCSEs | 1.235 | 0.900 to 1.693 | 0.191 |
| National vocational qualifications | 0.605*** | 0.457 to 0.802 | 0.000 |
| A-levels/AS-levels | 0.878 | 0.738 to 1.045 | 0.142 |
| Undergraduate degree | 1.213** | 1.065 to 1.380 | 0.004 |
| Master’s Degree | 1.366** | 1.124 to 1.660 | 0.002 |
| PhD | 1.958** | 1.216 to 3.152 | 0.006 |
| Other formal qualification | 1.231 | 0.516 to 2.936 | 0.640 |
| No religion | 1.015 | 0.916 to 1.124 | 0.775 |
| Christian | 1.188* | 1.039 to 1.358 | 0.012 |
| Buddhist | 1.262 | 0.233 to 0.352 | 0.509 |
| Hindu | 0.855 | 0.478 to 1.529 | 0.597 |
| Jewish | 0.476 | 0.095 to 2.388 | 0.367 |
| Muslim | 1.067 | 0.727 to 1.565 | 0.740 |
| Sikh | Sample too small | Sample too small | - |
| Other | 10.024 | 0.458 to 219.180 | 0.143 |

*statistically significant at p<0.05

Table SA1.4.2: Mean rating for ease of understanding of the information for different sub-groups

| **Variable** | **Leaflet** | **Video** | **P value*** |
| --- | --- | --- | --- |
| Male | 1.943 | 1.720 | 0.001 |
| Female | 1.785 | 1.498 | 0.000 |
| No previous children | 1.923 | 1.631 | 0.000 |
| Previous children | 1.798 | 1.587 | 0.004 |
| Age: 18 to 24 years | 2.138 | 1.818 | 0.097 |
| Age: 25 to 34 years | 1.847 | 1.584 | 0.003 |
| Age: 35 to 45 years | 1.812 | 1.584 | 0.000 |
| Currently pregnant | 2.105 | 1.531 | 0.011 |
| Previously had screening | 1.676 | 1.402 | 0.026 |
| No formal qualifications | 1.750 | 1.429 | 0.289 |
| 1 to 4 O-levels/GCSEs | 2.333 | 1.647 | 0.067 |
| 5+ O-levels/GCSEs | 1.659 | 1.757 | 0.530 |
| National vocational qualifications | 1.745 | 1.911 | 0.300 |
| A-levels/AS-levels | 1.809 | 1.615 | 0.029 |
| Undergraduate degree | 1.907 | 1.509 | 0.000 |
| Master’s Degree | 1.874 | 1.630 | 0.038 |
| PhD | 1.769 | 1.500 | 0.355 |
| Other formal qualification | 2.833 | 1.333 | 0.007 |
| No religion | 1.767 | 1.573 | 0.001 |
| Christian | 1.911 | 1.629 | 0.001 |
| Buddhist | 1.556 | 1.700 | 0.520 |
| Hindu | 2.125 | 2.000 | 0.643 |
| Jewish | 2.500 | 1.000 | 0.199 |
| Muslim | 2.308 | 1.625 | 0.025 |
| Sikh | 1.500 | 2.500 | 0.221 |
| Other | 2.200 | 1.500 | 0.434 |
