# Supplementary material for: Understanding the Impact of Different Modes of Information Provision on Preferences for a Newborn Bloodspot Screening Program in the United Kingdom
Source: MDM Policy Pract. 2024 Mar 4;9(1):23814683241232935. doi: 10.1177/23814683241232935 (PMC10913504; doi:10.1177/23814683241232935)
Supplement: sj-pdf-1-mpp-10.1177_23814683241232935 – Supplemental material for Understanding the Impact of Different Modes of Information Provision on Preferences for a Newborn Bloodspot Screening Program in the United Kingdom [file sj-pdf-1-mpp-10.1177_23814683241232935.pdf]

Start

Next

Intro

Making Choices About Newborn Bloodspot Screening

In this survey we will show you some background information about newborn bloodspot screening. We will then ask you some questions about your preferences for a newborn bloodspot screening programme in the NHS.

You can take part in this survey even if you do not have children or if you have children but they have not had newborn bloodspot screening

There are 5 phases to the survey

1. Background information on newborn bloodspot screening
2. What you thought about the background infromation
3. Completing the choice questions
4. Questions about your view of using information to make health care choices
5. Questions about you and if you have experience of the NHS newborn screening programme (you do not have to answer questions in this section if you do not wish to)

Back Next

0% 100%

PIS1

Taking part in this research

You are being invited to take part in a research study that aims to understand how information helps people to make decisions about newborn bloodspot screening. You will be provided with information about newborn bloodspot screening in the following pages. You do not have to have experience of screening to take part in this study.

Before you decide whether to take part in this study it is important for you to understand why the research is being done and what it will involve. The following page contains a summary of key information about the study. For full details about the study please click on the following link to download a copy of the full participant information sheet

[redacted for anonymisation]

Back Next

0% 100%

PIS2

Please take time to read the following information carefully. Take time to decide whether or not you wish to take part. Thank you for reading this.

What is the aim of the research?

This study wants to understand how medical information helps people make choices about healthcare. The aim is to explore how people feel about different aspects of newborn bloodspot screening.

Why have I been chosen?

This study aims to explore the views of members of the public. Anyone who is fluent in English is able to take part in this study.

What happens to the data collected?

The answers to the survey will be used to help us understand how people make decisions about their healthcare preferences and how information can be better provided to help this decision making. Your name will not be recorded and all survey answers will be given an anonymous identification number.

How is confidentiality maintained?

Laws called the Data Protection Act (2018) and General Data Protection Regulation (GDPR) tell us how to keep your information secure. All researchers are trained with this in mind, and your data will be looked after in the following way:

No information which would allow you to be identified will be collected by the research team. Data will be stored in secure [redacted for anonymisation] servers and computers. The data collected during this study will be stored for 5 years and then destroyed. The data collected in this study will not be shared outside of the members of the research team who are based at the University of Manchester.

Will the outcomes of the research be published?

The main outcome of this research will be a report presenting how different types of information affect people's decision making about healthcare programmes. In addition, we may want to report the findings at conferences or in a published journal article.

Contact details

If you have any queries about the study then please contact the researcher.

MR STUART WRIGHT, RESEARCH FELLOW IN HEALTH ECONOMICS

Email: stuart.j.wright@manchester.ac.uk

Telephone: 0161 306 7970

Do you consent to take part in this study?

PIS2=1 Yes

PIS2=2 No

Back Next

0% 100%

backgroundgender

We would now like to ask you some questions to determine your eligibility for this study

Q1: What gender do you most closely identify with?

backgroundgender=1 Male

backgroundgender=2 Female

children

Q2: How many children do you have?

children=1 None

children=2 One

children=3 Two

children=4 Three or more

Back Next

0% 100%

quota

[QUOTA: quota]

Back Next

Note:

This page only contains quota information and only appears in preview mode.

0% 100%

infointro

Thank you for agreeing to take part in this study

We will now show you some background information about an NHS screening programme called newborn bloodspot screening. After you have looked at this information we will ask you some questions about your preferences for newborn bloodspot screening

Please press next to continue

Back Next

0% 100%

leaflet1

Blood spot

RECOMMENDED

Purpose of screening

To find out if your baby has any of 9 rare but serious health conditions.

Early treatment can improve your baby's health and prevent severe disability or even death. If you, the baby's father, or a family member already has one of these conditions, please tell your health professional straight away.

Blood spot screening is recommended as it can save your baby's life

About these conditions

Sickle cell disease

About 1 in 2,800 babies born in the UK has a sickle cell disease (SCD). These are serious, inherited blood diseases. They affect haemoglobin, a part of the blood that carries oxygen around the body. Babies who have SCD will need specialist care throughout their lives.

People with SCD can have attacks of severe pain, get serious, life-threatening infections and are usually anaemic (their bodies have difficulty carrying oxygen). Babies with SCD can receive early treatment, including vaccinations and antibiotics, which, along with support from their parents, will help prevent serious illness and allow them to live a healthier life.

leaflet2

Cystic fibrosis

About 1 in 2,500 babies born in the UK has cystic fibrosis (CF). This inherited condition affects the digestion and lungs. Babies with CF may not gain weight well and frequently have chest infections.

Babies with CF can be treated early with a high-energy diet, medicines and physiotherapy. Although children with CF may still become very ill, early treatment can help them live longer, healthier lives.

Congenital hypothyroidism

About 1 in 2,000 babies born in the UK has congenital hypothyroidism (CHT). Babies with CHT do not have enough of the hormone thyroxine. Without thyroxine babies do not grow properly and they can develop permanent serious physical problems and learning disabilities.

Babies with CHT can be treated early with thyroxine tablets and this will allow them to develop normally.

Inherited metabolic diseases

It is important to let your health professional know if you have a family history of a

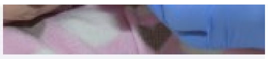

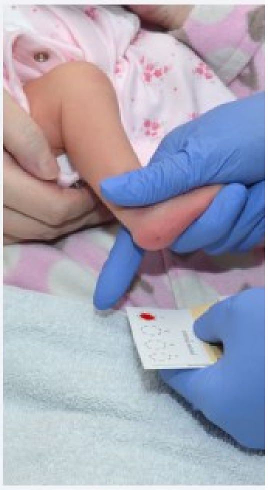

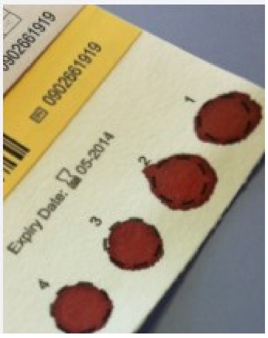

Back Next

0% 100%

leaflet3

metabolic condition. Babies are screened for 6 inherited metabolic diseases (IMDs).

These are:

- phenylketonuria (PKU)
- medium-chain acyl-CoA dehydrogenase deficiency (MCADD)
- maple syrup urine disease (MSUD)
- isovaleric acidaemia (IVA)
- glutaric aciduria type 1 (GA1)
- homocystinuria (pyridoxine unresponsive) (HCU)

About 1 in 10,000 babies born in the UK has PKU or MCADD. The other conditions are rarer, occurring in 1 in 150,000 babies to 1 in 300,000 babies.

Babies with these inherited conditions cannot process certain substances in their food. Without treatment babies with some of these conditions can become suddenly and seriously ill. The symptoms of the conditions are different; some may be life threatening or lead to severe developmental problems.

The information collected on your baby's blood spot card is important – make sure all the details are correct

They can all be treated by a carefully managed diet, which is different for each condition and may include additional medicines.

leaflet4

The screening test

When your baby is 5 days old the health professional will prick your baby's heel using a special device to collect some drops of blood onto a card. Occasionally this may be later than 5 days. The heel prick may be uncomfortable and your baby may cry. You can help by making sure your baby is warm and comfortable, and by cuddling and feeding them.

Sometimes, a second blood spot sample is required later on. If so, the reason will be explained. It does not necessarily mean there is something wrong with your baby.

Safety of the test

There are no known risks to your baby associated with having the test.

Screening is your choice

Screening your baby for all these conditions is recommended because it could save your baby's life but it is not compulsory. You can choose to have screening for SCD, CF or CHT individually but can only choose to have screening for all 6 IMDs or none at all. If you do not want your baby screened for any of the conditions or have any concerns about the test, please talk to your midwife.

Early screening is best as treatment can be started as soon as possible if needed. But if you choose not to have screening, your baby can have the test later if you change your mind. Babies can be screened up to 12 months of age for all the conditions except CF (only up to 8 weeks of age).

leaflet5

Possible results

Most babies will have normal results indicating that it is unlikely that they have any of the conditions. A small number of babies will screen positive for one of the conditions. This does not mean they have the condition but they are more likely to have it. They will be referred to a specialist for further tests.

Screening for cystic fibrosis finds some babies who may be genetic carriers of the condition. These babies may need further testing. Screening does not detect all carriers.

Occasionally, other medical conditions might be identified through these screening tests. For example, babies with beta thalassaemia major (a serious blood disease) will usually be detected. These children also need to be referred for lifelong treatment and care.

Screening for sickle cell disease also finds babies who are genetic carriers of these or other red blood cell diseases. Carriers are healthy although they can experience some problems in situations where their bodies might not get enough oxygen, for example, if they are having an anaesthetic.

leaflet6

Getting my results

You should receive the results from a health professional by the time your baby is 6 weeks old. The results should be recorded in your baby's personal child health record ('red book'). Please keep this safe and bring it with you to any further appointments. You will be contacted sooner if there is thought to be any problem with your baby.

My baby's blood spot card and data after screening

After screening blood spot cards are stored for at least 5 years and may be used:

- to check the result or for other tests recommended by your doctor
- to improve the screening programme
- for research to help improve the health of babies and their families in the UK

This research will not identify your baby and you will not be contacted. The use of these blood spots is governed by the code of practice available from your midwife, or on the website.

There is a small chance researchers may want to invite you or your child to take part in research linked to this screening programme. If you do not want to be invited to take part in research, please let your midwife know.

sciani

Please watch this video which contains information about Newborn Bloodspot Screening. The button to proceed to the next page will appear shortly

00:00 / 00:00

Back Next

0% 100%

infoeasy

Q3: Did you find this information easy or hard to understand?

infoeasy=1	infoeasy=2	infoeasy=3	infoeasy=4	infoeasy=5
1. Very easy	2. Quite easy	3. Neither easy or hard	4. Quite hard	5. Very hard

informedconsent

Q4: Imagine you were trying to decide whether your child should receive newborn bloodspot screening.

Do you think the information you have just been shown would make it easy or hard to make an informed choice about your child receiving newborn bloodspot screening?

informedconsent=1	informedconsent=2	informedconsent=3	informedconsent=4	informedconsent=5
1. Very easy	2. Quite easy	3. Neither easy or hard	4. Quite hard	5. Very hard

understand

Q5: Based on the information you were shown, was there anything you didn't understand about newborn bloodspot screening?

extrainfo

Q6: Is there any extra information that you would need to make an informed decision about newborn bloodspot screening?

Back Next

0% 100%

quintro

Your preferences for newborn bloodspot screening programmes

We will now ask you to complete seven questions about your preferences for newborn bloodspot screening. In each question you will be shown two potential newborn bloodspot screening programmes which vary in different ways. You will be asked to choose which newborn screening programme you would prefer to be offered by the NHS. You can also choose that no newborn screening programme be offered.

The newborn bloodspot screening programmes will have four characteristics:

The type of treatment available for the conditions screened for
Time to diagnosis and start of treatment
Is the blood sample stored?
Chance that the initial positive screening result is wrong

These characteristics and the potential levels they can take are explained in the next five pages

Back Next

0% 100%

treatment

Type of treatment available

The aim of newborn bloodspot screening programmes is to identify conditions early in a child's life so treatment can be given to potentially improve that child's length or quality of life. In the hypothetical screening programmes, treatment can have three potential effects for children identified with a condition:

Treatment will stop the disease from getting worse - This means that treatment will be able to stop the conditions from developing from a mild or moderate forms of the conditions into a more severe forms of the conditions.

Treatment will slow the worsening of the disease - This means that treatment will be able to slow the speed at which the conditions develop.

Treatment will relieve some of the symptoms of the disease - This means that treatment will stop some of the symptoms as a result of the conditions from occurring but will not slow the speed at which the conditions worsen or stop the conditions from getting worse.

ttr

Time to diagnosis and start of treatment

After a bloodspot is taken from a child, it will take time to send the sample to the laboratory, receive the results back and start treatment if the result is positive. In the hypothetical newborn bloodspot screening programmes, there are three potential lengths of time from the test to diagnosis and the start of treatment:

3 days from the time the test is taken to the start of treatment

7 days from the time the test is taken to the start of treatment

14 days from the time the test is taken to the start of treatment

21 days from the time the test is taken to the start of treatment

Back Next

0% 100%

bloodstore

Is the blood sample stored?

In the hypothetical newborn screening programmes, the bloodspot taken from a child's heel may be either stored or destroyed:

Yes - the bloodspot sample is stored and may be used for research purposes. No information that can be linked to the child is stored

No - the bloodspot is destroyed after the test has been completed

Back Next

0% 100%

falsepos

Chance that the initial positive screening result is wrong

Sometimes the newborn bloodspot screening test will suggest that a child may have a condition but further diagnostic tests actually show that they do not have the condition. This is sometimes known as a false-positive result and may cause anxiety for families. In the hypothetical screening programmes, children can have either a 1%, 2%, 5% or 10% chance of receiving a false-positive result. This chance is illustrated in the picture below and on the next page

This is an example of a screening programme with a false-positive risk of 1%

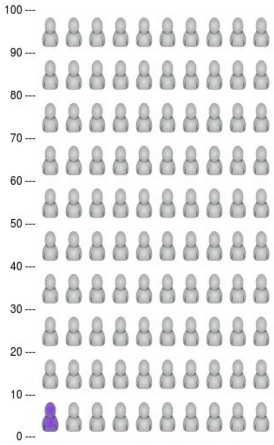

Back Next

0% 100%

FPexample1

This is an example of a screening programme with a false-positive risk of 10%

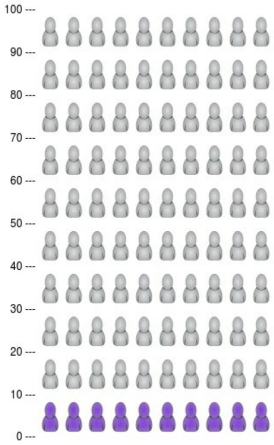

Back

Next

0% 100%

dceintro1

Making choices about newborn bloodspot screening

Now that you have seen the potential characteristics of the newborn bloodspot screening programmes, we will show you an example of a choice question. In this case the participant has chosen that they would prefer the first (far left) screening programme and not the second (middle) screening programme or for there to be no screening programme (far right).

	Screening Programme 1	Screening Programme 2	No Screening Programme
Severity of the conditions	Immediately life threatening	Life limiting	
Type of treatment available	Treatment will stop the disease from getting worse	Treatment will stop the disease from getting worse	
Time to diagnosis and start of treatment	14 days	7 days	
Is the blood sample stored?	Yes	No	There will be no chance of a false-positive result<br>No bloodspots will be stored<br>Disease will only be detected later in a child's life<br>The benefit of any treatment will be reduced
Chance that the initial positive screening result is wrong	1% 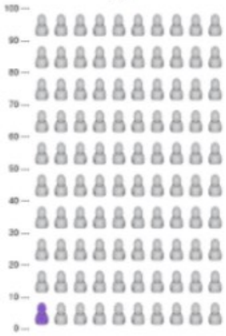	10% 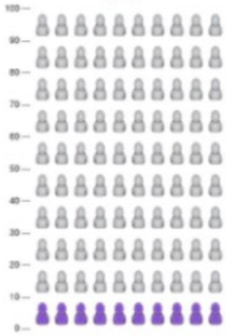	
	Select	Select	Select

We will now ask you to complete seven questions

Back Next

0% 100%

DCEblock1_Fixed1

Question 1 of 7: If you had to choose one of these screening programmes, each containing 9 conditions, which would you choose?

	Screening Programme 1	Screening Programme 2	
**Type of treatment available**	Treatment will stop the disease from getting worse	Treatment will slow the worsening of the disease	The benefit of any treatment will be reduced
**Time to diagnosis and start of treatment**	21 days	3 days	Disease will only be detected later in a child's life
**Is the blood sample stored?**	No	Yes	No bloodspots will be stored
**Chance that the initial positive screening result is wrong**	5% 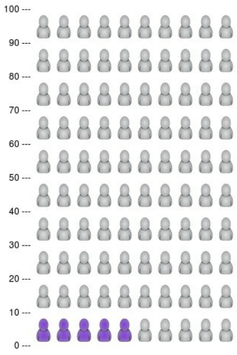	2% 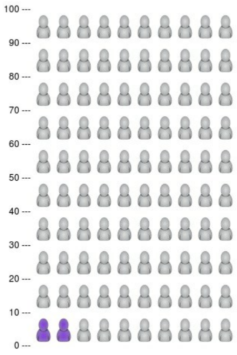	There will be no chance of a false-positive result
	DCEblock1_Fixed1 Select	DCEblock1_Fixed1 Select	DCEblock1_Fixed1 Select

Back Next

0% 100%

DCEblock1_Fixed2

Question 2 of 7: If you had to choose one of these screening programmes, each containing 9 conditions, which would you choose?

	Screening Programme 1	Screening Programme 2	
**Type of treatment available**	Treatment will relieve some of the symptoms of the disease	Treatment will slow the worsening of the disease	The benefit of any treatment will be reduced<br><br>Disease will only be detected later in a child's life
**Time to diagnosis and start of treatment**	14 days	7 days	No bloodspots will be stored
**Is the blood sample stored?**	Yes	No	There will be no chance of a false-positive result
**Chance that the initial positive screening result is wrong**	2%<br>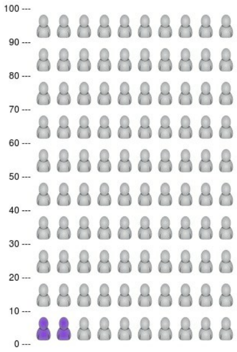	5%<br>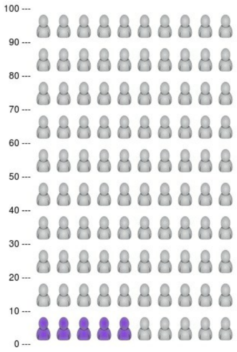	
	DCEblock1_Fixed2<br>Select	DCEblock1_Fixed2<br>Select	DCEblock1_Fixed2<br>Select

Back Next

0% 100%

DCEblock1_Fixed3

Question 3 of 7: If you had to choose one of these screening programmes, each containing 9 conditions, which would you choose?

	Screening Programme 1	Screening Programme 2	
**Type of treatment available**	Treatment will slow the worsening of the disease	Treatment will relieve some of the symptoms of the disease	The benefit of any treatment will be reduced<br><br>Disease will only be detected later in a child's life<br><br>No bloodspots will be stored<br><br>There will be no chance of a false-positive result
**Time to diagnosis and start of treatment**	3 days	21 days	
**Is the blood sample stored?**	No	Yes	
**Chance that the initial positive screening result is wrong**	5%<br>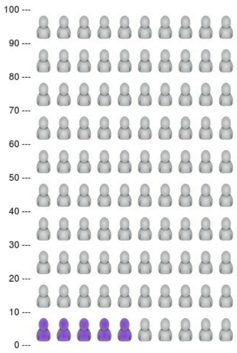	2%<br>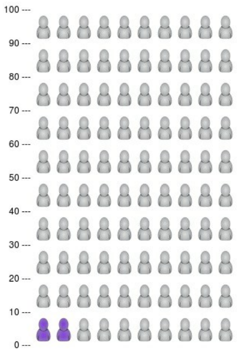	
	DCEblock1_Fixed3<br>Select	DCEblock1_Fixed3<br>Select	DCEblock1_Fixed3<br>Select

Back Next

0% 100%

DCEblock1_Fixed4

Question 4 of 7: If you had to choose one of these screening programmes, each containing 9 conditions, which would you choose?

	Screening Programme 1	Screening Programme 2	
**Type of treatment available**	Treatment will slow the worsening of the disease	Treatment will stop the disease from getting worse	The benefit of any treatment will be reduced
**Time to diagnosis and start of treatment**	7 days	14 days	Disease will only be detected later in a child's life
**Is the blood sample stored?**	Yes	No	No bloodspots will be stored
**Chance that the initial positive screening result is wrong**	1% 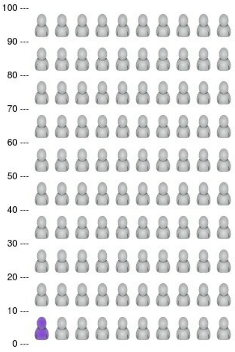	10% 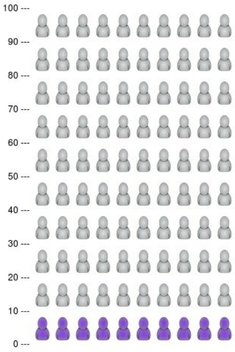	There will be no chance of a false-positive result
	DCEblock1_Fixed4 Select	DCEblock1_Fixed4 Select	DCEblock1_Fixed4 Select

Back Next

0% 100%

DCEblock1_Fixed5

Question 5 of 7: If you had to choose one of these screening programmes, each containing 9 conditions, which would you choose?

	Screening Programme 1	Screening Programme 2	
**Type of treatment available**	Treatment will stop the disease from getting worse	Treatment will stop the disease from getting worse	The benefit of any treatment will be reduced
**Time to diagnosis and start of treatment**	21 days	3 days	Disease will only be detected later in a child's life
**Is the blood sample stored?**	Yes	Yes	No bloodspots will be stored
**Chance that the initial positive screening result is wrong**	10% 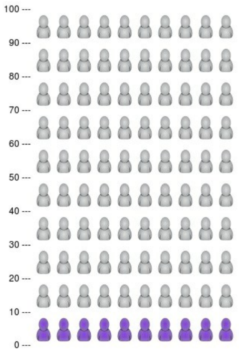	1% 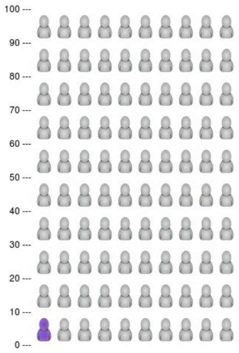	There will be no chance of a false-positive result
	DCEblock1_Fixed5 Select	DCEblock1_Fixed5 Select	DCEblock1_Fixed5 Select

Back Next

0% 100%

DCEblock1_Fixed6

Question 6 of 7: If you had to choose one of these screening programmes, each containing 9 conditions, which would you choose?

	Screening Programme 1	Screening Programme 2	
**Type of treatment available**	Treatment will relieve some of the symptoms of the disease	Treatment will slow the worsening of the disease	The benefit of any treatment will be reduced<br>Disease will only be detected later in a child's life<br>No bloodspots will be stored<br>There will be no chance of a false-positive result
**Time to diagnosis and start of treatment**	21 days	3 days	
**Is the blood sample stored?**	No	Yes	
**Chance that the initial positive screening result is wrong**	2% 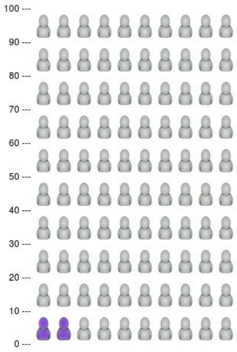	5% 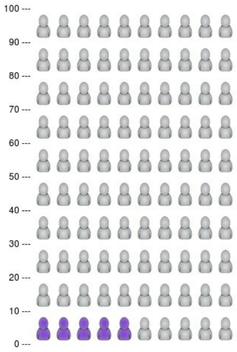	
	DCEblock1_Fixed6 Select	DCEblock1_Fixed6 Select	DCEblock1_Fixed6 Select

Back Next

0% 100%

DCEblock1_Fixed7

Question 7 of 7: If you had to choose one of these screening programmes, each containing 9 conditions, which would you choose?

	Screening Programme 1	Screening Programme 2	
**Type of treatment available**	Treatment will relieve some of the symptoms of the disease	Treatment will stop the disease from getting worse	The benefit of any treatment will be reduced<br>Disease will only be detected later in a child's life<br>No bloodspots will be stored
**Time to diagnosis and start of treatment**	7 days	14 days	
**Is the blood sample stored?**	Yes	No	There will be no chance of a false-positive result
**Chance that the initial positive screening result is wrong**	10%<br>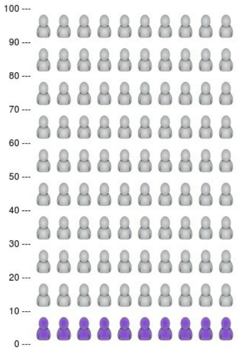	1%<br>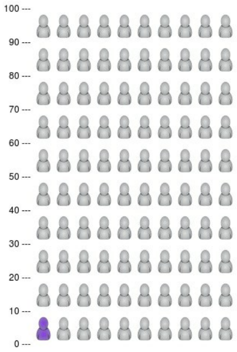	
	DCEblock1_Fixed7<br>Select	DCEblock1_Fixed7<br>Select	DCEblock1_Fixed7<br>Select

Back Next

0% 100%

check

We would now like to ask you some questions about how you found answering these questions

Q8: How confident are you that you would make the same choices if faced with the situations in real-life?

- check=1 Very confident I would make the same choices
- check=2 Quite confident I would make the same choices
- check=3 Not confident I would make the same choices
- check=4 Confident I would make different choices

difficulty

Q9: On a scale of 1 to 5, how easy or difficult did you find making choices between the alternatives?

difficulty=1	difficulty=2	difficulty=3	difficulty=4	difficulty=5
1 Very easy	2 Quite easy	3 Neither easy or difficult	4 Quite difficult	5 Very difficult

Back Next

0% 100%

nonattendance

Q10: Did you find yourself making choices based on one or two attributes in the choices?

nonattendance=1 Yes I focussed on one or two of the attributes

nonattendance=2 No I used all of the attributes to make my choices

Back Next

0% 100%

attributechoice

Q11: Which attributes did you use to make your choices?

attributechoice_1 Type of treatment available

attributechoice_2 Time to diagnosis and start of treatment

attributechoice_3 Is the blood sample stored?

attributechoice_4 Chance that the initial positive screening result is wrong

Back Next

0% 100%

informationpref

Q12: Think about the following statements in terms of how you react when you’re dealing with health concerns

Please mark how much you agree with each statement

	1 (strongly disagree)	2 (disagree)	3 (no opinion)	4 (agree)	5 (strongly agree)
I like to gather as much information as I can before I make a decision	informationpref_r1=1	informationpref_r1=2	informationpref_r1=3	informationpref_r1=4	informationpref_r1=5
I have difficulty making sense of information from multiple sources	informationpref_r2=1	informationpref_r2=2	informationpref_r2=3	informationpref_r2=4	informationpref_r2=5
I fear that I might find out something I don’t want to know	informationpref_r3=1	informationpref_r3=2	informationpref_r3=3	informationpref_r3=4	informationpref_r3=5
I like to review information multiple times before making a decision	informationpref_r4=1	informationpref_r4=2	informationpref_r4=3	informationpref_r4=4	informationpref_r4=5
I like to make decisions quickly	informationpref_r5=1	informationpref_r5=2	informationpref_r5=3	informationpref_r5=4	informationpref_r5=5
After I’ve made a decision, I continue to look for related information	informationpref_r6=1	informationpref_r6=2	informationpref_r6=3	informationpref_r6=4	informationpref_r6=5
I think it’s the doctor’s job to deal with information, not mine	informationpref_r7=1	informationpref_r7=2	informationpref_r7=3	informationpref_r7=4	informationpref_r7=5

Back Next

0% 100%

ethicview

Q13: In this question we would like to ask you some questions about your personal views

Please mark how much you agree with each statement

	1 (strongly disagree)	2 (disagree)	3 (no opinion)	4 (agree)	5 (strongly agree)
I would blame myself if my baby was unwell	ethicview_r1=1	ethicview_r1=2	ethicview_r1=3	ethicview_r1=4	ethicview_r1=5
My family would blame me if my baby was unwell	ethicview_r2=1	ethicview_r2=2	ethicview_r2=3	ethicview_r2=4	ethicview_r2=5
I think society is more hostile to disabled individuals	ethicview_r3=1	ethicview_r3=2	ethicview_r3=3	ethicview_r3=4	ethicview_r3=5
I think all bloodspot cards should be destroyed	ethicview_r4=1	ethicview_r4=2	ethicview_r4=3	ethicview_r4=4	ethicview_r4=5
I think bloodspot cards should be stored but used for scientific research only	ethicview_r5=1	ethicview_r5=2	ethicview_r5=3	ethicview_r5=4	ethicview_r5=5
I think bloodspot cards should be stored and used as a source of DNA in criminal cases	ethicview_r6=1	ethicview_r6=2	ethicview_r6=3	ethicview_r6=4	ethicview_r6=5

Back Next

0% 100%

age

We would now like to ask you some questions about yourself

Q14: Which age category do you fall into?

age=1 18-24

age=2 25-34

age=3 35-45

Back Next

0% 100%

education

Q15: What is the highest level of education you have obtained?

education=1 No formal qualifications

education=2 1-4 O levels/GCSEs

education=3 5+ O levels/GCSEs

education=4 National vocational qualifications (NVQs)

education=5 A-levels/AS-levels

education=6 Undergraduate degree

education=7 Master's degree

education=8 PhD

education=9 Other formal qualification

Back Next

0% 100%

Religion

Q16: What is your religion?

- Religion=1 No religion
- Religion=2 Christian
- Religion=3 Buddhist
- Religion=4 Hindu
- Religion=5 Jewish
- Religion=6 Muslim
- Religion=7 Sikh
- Religion=8 Religion_8_other Any other religion (please specify)

Back Next

0% 100%

pregnant

Q17: Are you or your partner currently pregnant?

pregnant=1 Yes

pregnant=2 No

pregnant=3 Don't know

previouspreg

Q18: How many previous pregnancies have you or your partner had?

previouspreg=1 None

previouspreg=2 One

previouspreg=3 Two

previouspreg=4 Three or more

Back Next

0% 100%

prevscreening

Q19: Have you ever been offered newborn bloodspot screening for your baby?

- prevscreening=1 Yes
- prevscreening=2 No
- prevscreening=3 Don't know

prevscreening2

Q20: Has/have your child/children had newborn bloospot screening?

- prevscreening2=1 Yes (all)
- prevscreening2=2 Some did and some did not
- prevscreening2=3 None did
- prevscreening2=4 Don't know

Back Next

0% 100%

famhistory

Q21: Are you aware whether anyone in your immediate family has been diagnosed with one of these conditions (mother, father, brothers, sisters, your own children)? (please tick all that apply, if none apply please press next)

famhistory_1 Phenylketonuria (PKU)

famhistory_2 Congenital hypothyroidism (CH)

famhistory_3 Cystic Fibrosis (CF)

famhistory_4 Sickle cell disease (SCD)

famhistory_5 Medium Chain Acyl Coenzyme-A Dehydrogenase Deficiency (MCADD)

famhistory_6 Maple Syrup Urine Disease (MSUD)

famhistory_7 Isovaleric Acidaemia (IVA)

famhistory_8 Glutaric Acidaemia Type 1 (GA1)

famhistory_9 Homocystinuria (HCU)

Back Next

0% 100%

concern

Q22: To what extent are you concerned about your offspring's risk of having one of these conditions?

concern=1 Very concerned

concern=2 Somewhat concerned

concern=3 Not very concerned

concern=4 Not concerned at all

Back Next

0% 100%

comments

Q23: Do you have any comments or feedback on this survey?
(Please include your feelings about the length of questions, terminology used, explanations provided *etc*)

Back Next

0% 100%

finish1

Thank you for taking part in this study

If you would like to find out more information about newborn bloodspot screening in the UK, please visit the following website:
https://www.nhs.uk/conditions/pregnancy-and-baby/newborn-blood-spot-test/

Back Next

0% 100%

finish

Note:

When respondents take the survey in regular mode this page will not be displayed. Respondents will be redirected to the link below:

https://dkr1.ssisurveys.com/projects/end?rst=1&psid= [Script] &basic=53677

0% 100%

disqualify

Note:

When respondents take the survey in regular mode this page will not be displayed. Respondents will be redirected to the link below:

https://dkr1.ssisurveys.com/projects/end?rst=2&psid= [Script]

0% 100%

endquota

Note:

When respondents take the survey in regular mode this page will not be displayed. Respondents will be redirected to the link below:

https://dkr1.ssisurveys.com/projects/end?rst=3&psid= [Script]

0% 100%
